# Positive Impact of Health Check-Ups and Guidance in the General Population: A Database-Based Cohort Study in Japan

**DOI:** 10.1016/j.focus.2025.100380

**Published:** 2025-06-17

**Authors:** Yukio Shimasaki, Masanori Nojima

**Affiliations:** 1 Graduate School of Frontier Science, The University of Tokyo, Kashiwa, Japan; 2 Center for Translational Research, The Institute of Medical Science Hospital, The University of Tokyo, Tokyo, Japan; 3 Division of Advanced Medicine Promotion, The Institute of Medical Science, The University of Tokyo, Tokyo, Japan

**Keywords:** Behavioral change, health check-up, health guidance, health insurance, Japan, metabolic syndrome

## Abstract

**Introduction:**

Despite ongoing debate, the effectiveness of health check-ups as a general population health approach has not been conclusively determined. This retrospective cohort study aimed to evaluate the effect of a health check-up program on various health parameters over a long-term period in accordance with a long-standing practice of health check-ups and guidance in Japan.

**Methods:**

Data from 3 prefectures in the Kanto region, encompassing individuals receiving health check-ups (aged 40–74 years) between 2008 and 2018, were obtained from the National Database of Health Insurance Claims and Specific Health Checkups of Japan. Health outcomes were tracked and analyzed using statistical models, stratifying by stages of behavioral change. The exposures of interest were health guidance (active support and motivational support) and whether or not health check-ups were skipped.

**Results:**

The analysis suggests that receiving active or motivational support was associated with reductions in metabolic syndrome indicators among participants with multiple visits (*n*=2,372,239). In the year after active support, risk reductions were observed for systolic blood pressure ≥130 mmHg (−17% in both sexes), triglycerides ≥150 mg/dL (−27% in both sexes), HbA1c ≥5.6% (−20.7% in men and −17.8% in women), BMI ≥25 (−41.2% and −49.3% in men and women, respectively). Although the magnitude of effect on each indicator was about half that of active support, receiving motivational support was similarly associated with improvements in most indicators. Analysis based on a fuzzy regression discontinuity design provides robustness of the results. When individuals missed health check-ups, the risks of hypertension and high triglycerides were increased by 5%–10% and 5%–20%, respectively. Significant associations were also observed with a reduced risk for the need for antihyperlipidemic medication as well as cardiovascular disease onset and either cerebrovascular or cardiovascular disease onset in men and a reduced risk of cerebrovascular disease onset in women after active support. In addition, undergoing regular check-ups—defined as receiving health check-ups at every opportunity versus 2 times or fewer within 5 years—was associated with a reduced risk of high blood pressure, high triglycerides, elevated HbA1c, and high BMI in both men and women, particularly in individuals covered by national healthcare insurance.

**Conclusions:**

Study findings suggest that the health guidance in the Japan’s specific health check-up programs in a general population is associated with improved health outcomes. Regular engagement also appears to be linked to sustained health benefits, highlighting the importance of strategies to enhance the long-term participation.

## HIGHLIGHTS

• There is a positive impact of Japan's specific health check-up program on metabolic syndrome.
• The benefits were observed regardless of the willingness for healthier lifestyles.
• This could influence the development of health initiatives in other countries.

## INTRODUCTION

Specific Health Check-ups and Health Guidance programs in Japan, established and in operation since 2008, aim to identify individuals with metabolic syndrome or those at risk of developing it and to then actively encourage behavioral changes to improve various health indicators and test results. Even prior to 2008, health check-up programs for workers were conducted for a long time, and in 1989, the current forms of health check-up programs that also included blood tests were adopted.

In an RCT on health screening conducted in Denmark,^1^ no significant effects were observed in reducing total mortality or cardiovascular events. Such findings may influence national decisions regarding the implementation of similar health check-ups. However, the content and frequency of the health check-ups and guidance in the Danish study were simpler than those of study conducted in Japan. Similarly, a meta-analysis of general health check-ups published in Cochrane Reviews in 2019 showed results comparable with those of the Danish RCT.^2^ However, the health check-up methods used in many trials reviewed differed from those in Japan; many were older studies initiated in the 1960s and 1970s. Although few similar studies have been conducted in Japan, several have shown positive results.^3^^,^^4^ Others have reported only a minor reduction in weight and BMI without significant changes in blood pressure, HbA1c, and low-density lipoprotein (LDL) with health check-ups and guidance.^5^ Overall, such results do not strongly support the effectiveness of health check-ups and guidance. However, to comprehensively evaluate the effectiveness of such programs, it is necessary to identify subgroups that are highly motivated to respond to guidance-based improvements. Owing to their limited sample sizes, previous studies only focused on an overall evaluation of participants, which was inadequate. With health check-up initiatives already widely expanded in Japan, it is necessary to conduct new studies that align with the actual conditions of health check-ups and health guidance.

In Japan, where annual workplace health examinations are the norm, doubts about the effectiveness of health check-ups are not currently considered a major issue. However, this does not diminish the importance of verifying their actual impact. In a context where health check-ups are already widely implemented, evaluating their effectiveness is both feasible and meaningful. This verification process can clarify their true value and provide important data for public health strategies. Although such an initiative could be unique to Japan, the question of whether regular health check-ups yield significant health benefits has broader global implications. Hence, the authors conducted this study in accordance with Japan’s health check-up and guidance practices, using anonymized health check-up data from the Database of Health Insurance Claims and Specific Health Checkups of Japan (NDB) provided by the Ministry of Health, Labour and Welfare. This nationwide database comprehensively covers insured individuals in Japan. In addition, this retrospective study aimed to evaluate the effect of the program on various health parameters over a long-term period with regard to participation in health check-ups and guidance.

## METHODS

### Study Population

Residents of 3 prefectures in the Kanto region (Saitama, Chiba, and Tokyo) who attended specific health check-ups at least once from 2008 to 2018 were selected from the NDB (Approval Number 1416).

Specific health guidance includes 2 types of support: Active Support and Motivational Support. The following are condition descriptions for health guidance assessment algorithm:

A. Obesity
 A1: Abdominal circumference ≥85 cm for men and ≥90 cm for women.
 A2: BMI of 25 or more (excluding A1).
B. Metabolic indicators
 B1: Systolic blood pressure ≥130 mmHg or diastolic blood pressure ≥85 mmHg.
 B2: Triglycerides ≥150 mg/dL or high-density lipoprotein (HDL) <40 mg/dL.
 B3: Fasting blood glucose ≥100 mg/dL or HbA1c ≥5.6%.
C. Smoking

For ages 40–64 years:

Under A1: Eligible for active support if meeting any 2 B or 1 B plus C; eligible for motivational support if only 1 B is met.
Under A2: Eligible for active support if meeting all B or any 2 B plus C; eligible for motivational support if meeting any 2 B without C or just 1 B.

For ages ≥65 years, under either A1 or A2: Always eligible for motivational support.

If undergoing medication therapy for 1 of the B, participants were ineligible for any health guidance. Motivational and active supports in exercise, diet, and medical visits were provided, either face to face or online, by trained instructors who were supervised by physicians, public health nurses, and dietitians. For motivational support, a single interview with the instructor is conducted, followed by a follow-up evaluation 3–6 months after the initial guidance. For active support, continuous assistance is provided for over 3 months—through in-person meetings, phone calls, or letters—after the initial interview, with an evaluation 3–6 months after guidance. Appendix Figure 1 (available online) summarizes participant selection for each cohort. Appendix Text (available online) provides further details, including the definition of each cohort.

### Measures

The authors included all participants who completed health check-ups, consisting of (1) questionnaires covering medication use, past history of cerebrovascular or cardiovascular disease (CVD), and the stage of behavioral change; (2) physical examinations (height, weight, abdominal circumference, and blood pressure); (3) laboratory tests (serum lipids, liver function tests, blood glucose, and HbA1c); and (4) urinalyses. Data regarding the type of health insurance, including national health insurance (NHI) (mainly for self-employed individuals) and employee health insurance (EHI), were only available from 2013. The stages of behavioral change were defined as follows: Stage 1, no intention to improve; Stage 2, intends to improve within 6 months; Stage 3, intends to improve within 1 month; Stage 4, already working on improvement (<6 months); and Stage 5, already working on improvement (≥6 months). Further details are provided in Appendix Text (available online).

### Statistical Analysis

Longitudinal changes in metabolic indicators were analyzed using a linear mixed model, comparing participants who received health guidance with those who did not, with/without an interaction term between health guidance status and time. The outcomes were the indicator values measured at check-ups in the following years. To assess differences and adjust for the willingness to improve lifestyle habits, the authors stratified analyses by stages of behavioral change. Factors such as sex, age, BMI, smoking, drinking, and baseline values were also adjusted. Analyses using a fuzzy regression discontinuity design (fRDD) were conducted to explore the unbiased effect of health guidance as a sensitivity analysis at the cut offs of abdominal circumference. Binary outcomes for starting new medications (status changing from no to yes), the onset of diseases (history status changing from no to yes), and the indicators exceeding cut offs were assessed using a generalized estimation equation with an independent working correlation matrix. Subjects for the analysis of binary outcomes were free from the outcomes at the baseline. The authors assessed how skipping health check-ups impacted outcomes by comparing changes from the initial check-up to the 2-year follow-up between participants who missed and those who attended their intermediate year's check-ups. The authors analyzed the frequency of health check-up attendance and laboratory test exceedances using generalized estimation equation. All analyses were performed in R 4.2.2 (R Core Team, Vienna, Austria) with the lme4, gee, and rdrobust packages, considering a *p*<0.05 as statistically significant.

This study was approved by the ethics review committee of the Institute of Medical Science, the University of Tokyo (Approval Number 2020-29-0902). Data obtained from the NDB are restricted to those who have submitted a data-use application.

## RESULTS

Appendix Figure 1 (available online) is an overview of study participants. Appendix Tables 1–3 (available online) present the demographic characteristics of study participants and crude statistics of health check-up outcomes in each cohort.

### The Effectiveness of Specific Health Guidance on Clinical Examination Items

**Figure 1:**
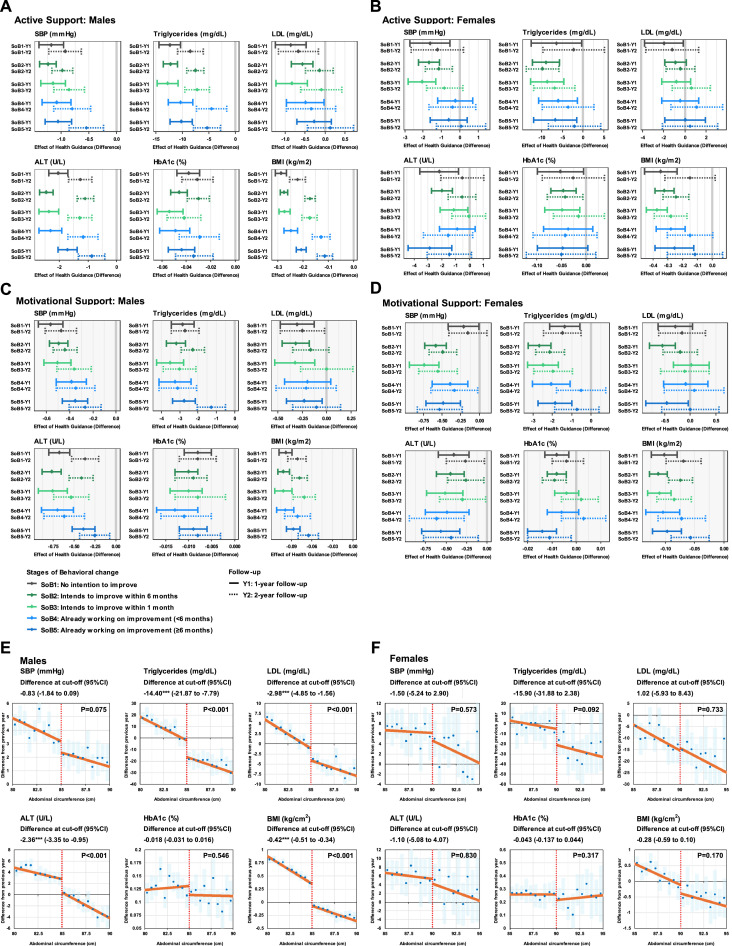
Effectiveness of health guidance on changes in metabolic indicators. This figure shows the model-based effectiveness of receiving health guidance among eligible participants stratified according to stage of behavioral change. Differences in continuous outcomes between those who received support and those who did not after 1- and 2-year follow-ups are shown by solid and dotted lines, respectively. Additional analyses using fRDD was also performed to explore the unbiased effect of being eligible for specific health guidance, regardless of whether it was received. (A, B) Changes in metabolic indicators for (A) males and (B) females who received active support. (C, D) The outcomes for (C) males and (D) females who received motivational support. At the subsequent visit, the effects were diminished, but the reduction was smaller than when active support was given. Regarding BMI, the reduction from the next visit to the subsequent visit for active support was from −49% to −23% for men and from −57% to −24% for women, whereas for motivational support, it was from −25% to −17% for men and from −41% to −23% for women. (E, F) Plots of the analyses based on fRDD at the cut offs of abdominal circumference for determining the eligibility for the health guidance in (E) males and (F) females. Robust CIs were employed (main/bias bandwidth=7.5/10, kernel=triangular). The Y-axis values were adjusted from those generated by conventional RDD, applying weights on the basis of the inverse probability of receiving the health guidance, as estimated by fRDD. ALT, alanine aminotransferase; fRDD, fuzzy regression discontinuity design; LDL, low-density lipoprotein; RDD, regression discontinuity design; SBP, systolic blood pressure; SoB, stage of behavioral change.

The effectiveness of specific health guidance on clinical examination items was measured. The actual percentage of those who received active support (Cohort 1) was 19.1% in men and 14.9% in women. As shown in Figure 1A and B and Appendix Figure 2A and B (available online), those who received support showed reductions in weight and BMI compared with those who did not, regardless of the stage of behavioral change (BMI changes were from −0.20 to −0.29 in men and −0.26 to −0.37 in women). In addition, in men, receiving support was associated with improvements in most indicators at the next visit. At the subsequent visit, the effect was reduced compared with that measured at the next visit, indicating potential difficulty in maintaining the observed improvements. Although marked differences between stages were not noted, the effect was slightly greater in Stages 1–3 and slightly lower in Stages 4 and 5, when behavioral changes had already begun. Similar trends were observed in women.

The actual percentage of those who received motivational support (Cohort 2) was 24.4% for men and 22.2% for women. Figure 1, Figure 1 and Appendix Figure 2C and D (available online) show that in both men and women, those who received support displayed reductions in weight and BMI compared with those who did not receive support, regardless of the stage of behavioral change (BMI changes were from −0.09 to –0.10 in men and –0.10 to –0.11 in women). Although the magnitude of effect on each indicator was about half that of active support, receiving motivational support was similarly associated with improvements in most indicators. Women showed similar trends (Figure 1D). Similar to that in active support, an effect of health guidance in men was observed for most indicators at all stages of behavioral change, with higher effects in Stages 1–3 and slightly lower effects in Stages 4 and 5.

The authors then conducted analyses using an fRDD to explore the unbiased effect of specific health guidance. As illustrated in Figure 1, Figure 1, statistically significant effects were observed in men for triglycerides, LDL, alanine aminotransferase, and BMI. Despite the unstable estimations due to limited number of samples near the cut off, similar trends were also observed in women. Effect sizes were comparable with the results in the regression analyses (Figure 1, Figure 1).

**Figure 2:**
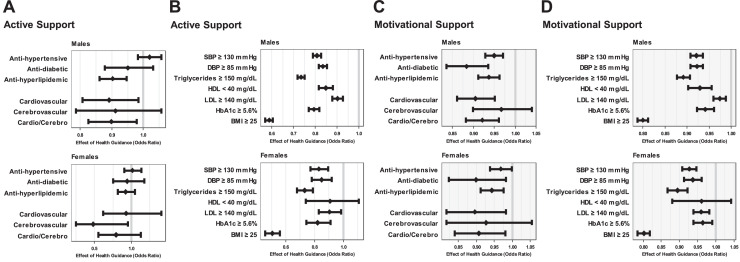
Effect of health guidance on new medications and exceeding certain cut offs of metabolic indicators. This figure shows model-based effectiveness of receiving health guidance among eligible participants. ORs are shown for binary outcomes between those who received support and those who did not after a 1-year follow-up. Owing to issues with the stability of the analysis, the stages of behavioral change were included as adjustment factors in models. (A, B) Effect of active support on the risk of (A) new medication/new onset of cardiocerebrovascular diseases and (B) exceeding certain cut offs of metabolic indicators. The risk of SBP ≥130 mmHg and DBP ≥85 mmHg decreased by 16.6% and 19.3% in men, respectively, and by 16.9% and 15.2% in women, respectively. The risk of triglycerides ≥150 mg/dL decreased by 27% in both men and women (26.6% and 26.9%), LDL ≥140 mg/dL decreased by 10% in both men and women (9.9% and 9.5%), and HbA1c ≥5.6% decreased by 20.7% in men and 17.8% in women. The risk of a BMI ≥25 decreased significantly by 41.2% in men and 49.3% in women. (C, D) Effect of motivational support on the risk of (C) receiving new medication/new onset of cardiocerebrovascular diseases and (D) exceeding certain cut offs of metabolic indicators. The risk of SBP ≥130 mmHg and DBP ≥85 mmHg decreased by 7.9% and 7.8% in men and by 7.3% and 6.3% in women. The risk of triglycerides ≥150 mg/dL decreased by around 10% in both men and women (10.9% and 10.5%), LDL ≥140 mg/dL decreased by 2.6% in men and by 4.0% in women, and HbA1c ≥5.6% decreased by 5.9% in men and 3.6% in women. The risk of BMI ≥25 decreased by 20% in both men and women (20.0% and 19.8%). DBP, diastolic blood pressure; HDL, higher-density lipoprotein; LDL, low-density lipoprotein; SBP, systolic blood pressure; SoB, stage of behavioral change.

The effectiveness of specific guidance on new medication and disease onset was also measured. In Cohort 1, significant associations were observed with a reduced risk for the need for antihyperlipidemic medication as well as CVD onset and either cerebrovascular or CVD onset in men (Figure 2A). A significant association was also observed with a reduced risk of cerebrovascular disease onset in women. Furthermore, when evaluating the risk of indicators exceeding certain cut offs based on a clinical consensus, the association with risk reduction was more clearly demonstrated (Figure 2B). For example, the risk of a BMI ≥25 decreased significantly by 41.2% in men and 49.3% in women.

In Cohort 2, ignificant associations were observed with a reduced risk for the need of antihypertensive, antidiabetic, and antihyperlipidemic medications; CVD onset; and either cerebrovascular or CVD onset in men (Figure 2C). Similar associations were found in women. Regarding the risk of metabolic indicators exceeding certain values, a clear association with risk reduction was demonstrated, similar to that observed with active support (Figure 2D). For example, the risk of BMI ≥25 decreased by 20% in both men and women (20.0% and 19.8%).

**Figure 3:**
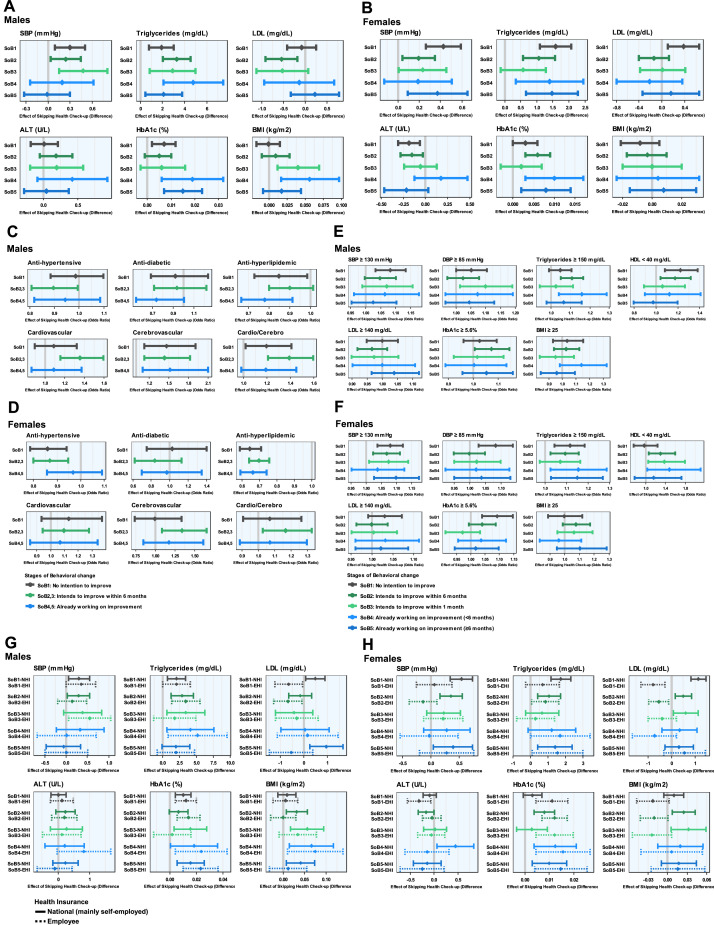
Effect of skipping health check-ups on new medications and exceeding certain cut offs of metabolic indicators. This figure shows model-based effectiveness of skipping health check-ups among ineligible healthy participants stratified by stage of behavioral change. (A, B, G, H) Differences in continuous outcomes. (C–F) ORs for binary outcomes at a health check-up 2 years later. The comparison was between participants who skipped their health check-up in the intervening year and those who did not. (A, B) Changes in metabolic indicators for (A) males and (B) females. Higher SBP (by 0.2–0.5 mmHg), higher triglycerides (by 2–5 mg/dL in men and 0.5–1.5 mg/dL in women), and higher HbA1c levels (by 0–0.02% in men and 0–0.01% in women) were observed in the group that skipped check-ups. (C, D) Effect on the risk of new medications/new onset of cardi-cerebrovascular diseases for (C) males and (D) females. (E, F) Effect on the risk of exceeding certain cut offs for metabolic indicators for (E) males and (F) females. (G, H) Changes in metabolic indicators stratified by the type of health insurance for (G) males and (H) females. Compared with A and B, the effects of the skipping health check-up on LDL and BMI were much clearer in the NHI group. ALT, alanine aminotransferase; DBP, diastolic blood pressure; EHI, employee health insurance; HDL, higher-density lipoprotein; LDL, low-density lipoprotein; NHI, national health insurance; SBP, systolic blood pressure; SoB, stage of behavioral change.

The authors examined the effect on survey items of skipping (not undergoing) health check-ups at subsequent visits in participants who were ineligible for health guidance and who had no history of receiving medication or a medical history (Cohort 3) (Figure 3; Appendix Figure 3, available online; and Appendix Table 3, available online). As a result, the skipping check-ups were associated with higher systolic blood pressure, triglycerides, and HbA1c in the following year (Figure 3, Figure 3). In addition, participants tended not to be prescribed antihypertensive or lipid-lowering medications when missing health check-ups (Figure 3, Figure 3). Although this appears contradictory—given that health guidance also tends to reduce prescriptions—skipping check-ups may result in missed opportunities to detect diseases, ultimately leading to a lack of necessary medication. This is consistent with an increase in hypertension risk shown in Figure 3, Figure 3, which was observed particularly in women and some men, where the risk increased by 5%–10% across various stages of behavioral change. In addition, an increase in the risk of high triglycerides was observed, which ranged from 5% to 20% in women and in some men. A tendency for the risk of cardiovascular and cerebrovascular disease onset to increase was also observed.

Furthermore, the authors also examined the differences in the effect of skipping health check-ups between the insured individuals of NHI (mainly for self-employed) and EHI, which showed significant disparities in health examination attendance rates (37.5% vs 68.7%, according to the report from Ministry of Health, Labour and Welfare 2022, https://www.mhlw.go.jp/stf/seisakunitsuite/bunya/newpage_00045.html), to assess its potential confounding and effect modification. The high attendance rate in EHI is due to strong encouragement from insurers. Results from Appendix Figure 3C and D (available online) show a higher risk of cerebrovascular disease in EHI during nonattendance periods. Figure 3, Figure 3 indicate that the effect of skipping clinical tests is much clearer in NHI. In contrast, as shown in Appendix Figure 3E and F (available online), there is no obvious difference in the effect of the health guidance between the types of the health insurance.

The authors examined the effects of repeated specific health guidance for individuals (Appendix Figures 4 and 5, available online) in Cohorts 1 and 2. In the case of active support for the second time, the effect on BMI was reduced by approximately 40% in men and 70% in women. For motivational support, the reduction was 32% in men and 41% in women (averaged across stages of behavioral change). Although the effect of guidance diminishes when it is repeated, as seen with BMI, the effect is not entirely lost.

**Figure 4:**
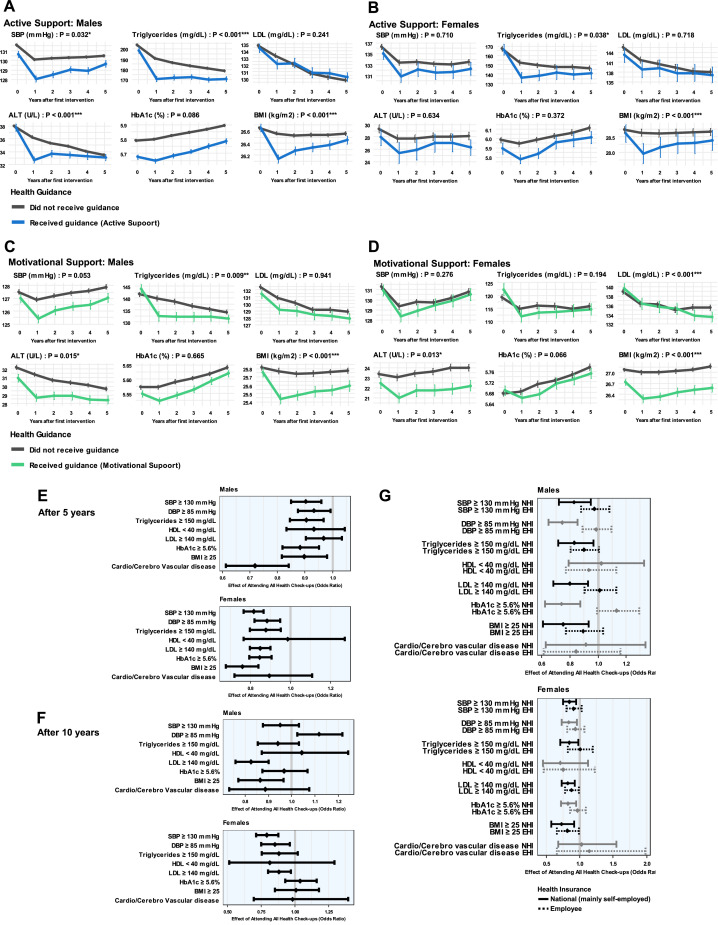
Effect of health guidance over time. This figure shows the longitudinal tracking of health outcomes comparing participants who received health guidance with those who did not. (A, B) Changes in metabolic indicators over 5 years for (A) males and (B) females who received active support. (C, D) Outcomes for (C) males and (D) females who received motivational support. Models used in these assessments included an interaction term between health guidance status and time. *p*-values were calculated for the interaction term using a linear mixed model, with adjustment for baseline values of the outcomes. Error bars represent 95% CIs for means. (E, F) Effect of regular health check-ups over 5 years ([E] attending health check-ups at every opportunity versus 2 times or fewer) and 10 years ([F] attending health check-ups at every opportunity versus 5 times or fewer) on the risk of exceeding certain cut offs of metabolic indicators and the new onset of cardiocerebrovascular diseases. (G) Subgroup analyses assessing the effect of regular health check-ups over 5 years, stratified by the type of health insurance. In the 5-year comparison, regular check-ups were associated with a reduced risk of high blood pressure (systolic: 9.7% in men and 18.4% in women, diastolic: 6.7% and 11.7%), high triglycerides (9.5% and 12.3%), high HbA1c (11.8% and 15.3%), and high BMI (10.3% and 24.0%) in men and women. A reduced risk of new-onset cardiovascular or cerebrovascular diseases was also observed in men (28.1%), and a reduced risk of high LDL was observed in women (15.2%) (in E). In the 10-year comparison, regular check-ups were associated with a reduced risk of high LDL (17.4%) and BMI (13.5%) in men and a reduced risk of high blood pressure (systolic: 21.0%, diastolic: 15.0%) and high LDL (11.9%) in women (in F). ALT, alanine aminotransferase; EHI, employee health insurance; LDL, low-density lipoprotein; NHI, national health insurance; SBP, systolic blood pressure.

Finally, the authors stratified participants eligible for health guidance according to whether they received guidance and examined the progression of various measurements over 5 years (Cohorts 4A and 5A, Figure 4A–D and Appendix Figure 6, available online). The effect of guidance resulted in significant improvements in some indicators the following year, which gradually diminished over time. Several indicators showed long-term differences between cohorts such as lower BMI in those who received health guidance. A 10-year examination (Cohorts 4B and 5B, Appendix Figure 7, available online) indicated that differences tended to diminish after 5 years. This analysis stratified participants solely by the initial receipt of health guidance and did not consider subsequent guidance, which may have contributed to the diminishing differences observed.

Furthermore, comparisons were made between those who received health check-ups 2 times or fewer within 5 years and those who received them at every opportunity. Comparisons were also made between those who received check-ups 5 times or fewer within 10 years and those who received them when offered (Cohorts 6A and 6B, Figure 4E [5 years later] and F [10 years later]). In the 5-year comparison, regular check-ups were associated with a reduced risk of high blood pressure, high triglycerides, high HbA1c, and high BMI in men and women. A reduced risk of new-onset cardiovascular or cerebrovascular diseases was also observed in men, and a reduced risk of high LDL was observed in women. In the 10-year comparison, regular check-ups were associated with a reduced risk of high LDL and BMI in men and a reduced risk of high blood pressure and high LDL in women. Furthermore, the authors also conducted subgroup analyses for NHI and EHI (Figure 4G). The results showed that in NHI, there was a stronger association between regular check-ups and a reduction in risk for each indicator.

## DISCUSSION

The effectiveness of health check-ups as a population health approach has not yet been conclusively determined despite several investigations. For example, the most impactful study showing negative results from community health check-ups is an RCT from Denmark.^1^ This study involved a program where participants underwent baseline health check-up and 1–3 following check-ups (for low risk and high risk, respectively) over 5 years with additional 5-year follow-up. However, the study found no improvements in reducing the incidence of ischemic heart disease, stroke, or overall mortality. One major issue with that study was the low participation rate in the intervention group (52.5%),^6^ resulting in a comparison between groups with significant baseline differences (e.g., for higher BMI, higher alcohol consumption, and lower smoking rates). This may have impaired the efficiency of the randomization. In the intervention group, participation in the intensive program was only about one third in the first and third years—a marked contrast to Japan's annual program, where 78.2% of health check-up participants underwent next year’s check-up, as calculated from the data. In Japan, health check-ups are conducted annually, providing repeated intervention opportunities. Even the second or third guidance can be expected to have some effect (Appendix Figures 4 and 5, available online). The intervention program of the Danish RCT, especially in terms of check-up frequency, differed from that of the Japanese program. In addition, the Danish study included very few blood test items (serum lipid profile only). Another Danish RCT (DANCAVAS) also involved a single intervention followed by a 5-year follow-up,^7^ again differing from the Japanese program. This second Danish study concluded that there were no significant effects overall but suggested potential efficacy in certain subgroups, such as for primary outcomes such as death from any cause for ages 65–69 years and stroke outcomes, despite issues with statistical multiplicity.

Several other observational studies in Japan have suggested the effectiveness of health guidance in improving health outcomes, similar to the findings of this study.^3^^,^^4^ Studies on the national health check-up program in Korea have indicated a correlation between the number of health check-ups and mortality rates,^8^ and regular attendance at health check-ups was associated with fewer CVD-related risk factors.^9^ Similarly, United Kingdom observational studies suggested effectiveness of NHS (National Health Service) health checkup.^10^^,^^11^ Because the attendance rates and frequency of health check-ups in these countries differ from those in Japan—approximately 60% in Korea (every 2 years) and 40% in the United Kingdom (every 5 years)^9^^,^^12^—caution should be exercised when extrapolating the results, although the situation in Korea is similar to that in Japan. As shown in Appendix Figures 4 and 5 (available online), the effect of health guidance appears to diminish with repeated interventions, suggesting that the effect observed in this study may be underestimated when considering it as a single intervention. In contrast to these reports, a recent Japanese study using the NDB examined the effect of notification for eligibility of health guidance (regardless of actual guidance received) using a regression discontinuity design and reported only a slight effect.^13^ This may be due to the limited effect of the notification of being eligible for the health guidance and the low receipt rate of the guidance demonstrated also in this study. In addition, the health check-up itself could promote health (as shown in Figure 3, Figure 4), making the notification effect limited.

From the perspective of subgroup differences, no clear variation in the effect of health guidance was observed on the basis of the stage of behavior change, although the effect tended to be slightly greater in Stages 1–3 and slightly lower in Stages 4 and 5. An effect was also observed even in groups presumed to have low motivation for health improvement (e.g., those with no intention to improve). In addition, as shown in Appendix Figure 3C–F (available online) and Figure 4G, the effectiveness of health check-ups appears to be greater among the NHI (mainly for self-employed) group, suggesting potential benefits in promoting attendance among these populations. The differences in the effects of skipping and regularly attending health check-ups between individuals with NHI and EHI are noteworthy. In EHI, those who skip health check-ups often suffer from serious conditions such as cerebrovascular diseases (Appendix Figure 3C, available online), but the benefits of continuous check-ups seem limited. This may be because insurers’ strong recommendations for check-ups prevent skips owing to simple motivational issues, leading to an enrichment of severe cases among those who do skip. As a result, for those with severe conditions, metabolic indicators can be influenced through medical supervision or conditions themselves. In contrast, individuals with NHI show an opposite trend. Given that the stages of behavior change and baseline conditions are included as adjusting factors, the observed effects in NHI participants could be linked to their motivation, maintained through their ongoing participation in check-ups. Interestingly, the report from Korea also indicated that the effect of health check-ups was greater in the self-employed population, with particularly stronger effects observed in the low-income group.^8^

The authors further assessed the significance of the measured items as risk factors for CVDs and overall mortality. High BMI has been associated with these health risks across numerous studies, regardless of race or sex,^14^, ^15^, ^16^, ^17^, ^18^, ^19^ and in Japanese populations, the lowest risk of all-cause mortality is reported to be in individuals with BMIs between 22.0 and 24.9 kg/m^2^.^20^ High triglycerides, which showed particularly significant effects from health guidance (Figure 1, Figure 2, Figure 3, Figure 4), are suggested as a risk factor for vascular events, independent of LDL and other factors.^21^, ^22^, ^23^ A report from East Asia indicates that a combination of an abdominal circumference >85 cm and triglyceride levels at 1.5 mmol/L (approximately 134 mg/dL) further increases the risk.^24^ In addition, these factors have been linked to rising healthcare costs.^25^ Similarly, for HbA1c, which also demonstrated a high effect (Figure 1, Figure 2, Figure 3, Figure 4), reports suggest that the lowest mortality risk is between 5.0% and 6.0% even in nondiabetics,^26^ with a linear correlation between HbA1c levels and cardiovascular event risks in nondiabetics reported.^27^ For HDL, which also indicated a relatively high effect from health guidance (Appendix Figures 2 and 3, available online, and Figure 2, Figure 3), there is a consensus that low HDL levels are associated with increased risks of cardiovascular events and all-cause mortality.^28^, ^29^, ^30^ The presence of risk-reduction effects for these 3 test items can be linked to the advantages of Japan's specific health examinations.

### Limitations

The limitations of this study include the inability to evaluate mortality, which was restricted by the nature of the database. Further limitations are detailed below. First, the use of medication and the onset of cardiocerebrovascular diseases as a clinical or mortality surrogate remain ambiguous without a direct link to a medical database. It is also possible that participants skipped check-ups because they were hospitalized or in need of medical care owing to the onset of CVDs or cerebrovascular diseases, so an interpretation should be approached with caution. Second, being an observational study, it was impossible to ascertain causal relationships. Nevertheless, by comparing whether eligible individuals received health guidance with not receiving, the authors aimed to standardize the comparison groups. In addition, by further stratifying these groups on the basis of their willingness to improve their lifestyle, a confounding effect could be minimized. The results based on fRDD also corroborate primary findings, demonstrating consistent effects of health guidance. Third, although the follow-up rate was not low for a health check-up continuity (78.2%), it was low for a cohort study. However, because the reasons for loss of follow-up may be linked to worsening conditions such as hospitalization, death, or a lack of awareness of health improvement, the estimated effects could be conservative. Fourth, the accuracy of effect estimates in women suffered from the smaller pool of eligible participants on the basis of the criteria used. In women, owing to an algorithmic issue in selecting individuals for health guidance, the number of participants was much lower than in men (approximately 1 to 10), making changes less clear, although similar trends were observed. It might be beneficial to revise the criteria for women to enhance their eligibility for health guidance. The low percentage of health guidance recipients, ranging from 10% to 20% for both sexes, needs improvement. Finally, although numerous outcomes showed significant effects, the magnitude of these effects must be weighed against the cost-effectiveness of the programs.

## CONCLUSIONS

This study demonstrated the effectiveness of Japan's specific health check-up and health guidance programs across various parameters. Indications are that such programs, which are free for participants (insured individuals), have a positive effect on the health management of a broad demographic of the Japanese population.

## Appendix Supplementary materials

Image, application 1

Image, application 2

Image, application 3

## Footnotes

Supplementary material associated with this article can be found in the online version at doi:10.1016/j.focus.2025.100380.

## References

[1] Jørgensen T., Jacobsen R.K., Toft U., Aadahl M., Glümer C., Pisinger C. (2014). Effect of screening and lifestyle counselling on incidence of ischaemic heart disease in general population: Inter99 randomised trial. BMJ.

[2] Krogsbøll L.T., Jørgensen K.J., Grønhøj Larsen C., Gøtzsche P.C. (2012). General health checks in adults for reducing morbidity and mortality from disease. Cochrane Database Syst Rev.

[3] Tsushita K., S Hosler A., Miura K. (2018). Rationale and descriptive analysis of specific health guidance: the nationwide lifestyle intervention program targeting metabolic syndrome in Japan. J Atheroscler Thromb.

[4] Nakao Y.M., Miyamoto Y., Ueshima K. (2018). Effectiveness of nationwide screening and lifestyle intervention for abdominal obesity and cardiometabolic risks in Japan: the metabolic syndrome and comprehensive lifestyle intervention study on nationwide database in Japan (MetS ACTION-J study). PLoS One.

[5] Fukuma S., Iizuka T., Ikenoue T., Tsugawa Y. (2020). Association of the national health guidance intervention for obesity and cardiovascular risks with health outcomes among Japanese men. JAMA Intern Med.

[6] Jørgensen T., Borch-Johnsen K., Thomsen T.F., Ibsen H., Glümer C., Pisinger C. (2003). A randomized non-pharmacological intervention study for prevention of ischaemic heart disease: baseline results Inter99. Eur J Cardiovasc Prev Rehabil.

[7] Lindholt J.S., Søgaard R., Rasmussen L.M. (2022). Five-year outcomes of the Danish cardiovascular screening (DANCAVAS) trial. N Engl J Med.

[8] Yun B., Oh J., Choi J. (2023). Socioeconomic disparities in the association between all-cause mortality and health check-up participation among healthy middle-aged workers: a nationwide study. J Korean Med Sci.

[9] Park B.H., Lee B.K., Ahn J., Kim N.S., Park J., Kim Y. (2021). Association of participation in health check-ups with risk factors for cardiovascular diseases. J Korean Med Sci.

[10] Artac M., Dalton A.R.H., Majeed A., Car J., Millett CJ. (2013). Effectiveness of a national cardiovascular disease risk assessment program (NHS Health Check): results after one year. Prev Med.

[11] McCracken C., Raisi-Estabragh Z., Szabo L. (2024). NHS Health Check attendance is associated with reduced multiorgan disease risk: a matched cohort study in the UK Biobank. BMC Med.

[12] NHS Health Check Programme Review, 2021, Office for Health Improvement and Disparities. Available from: https://www.gov.uk/government/publications/nhs-health-check-programme-review. Accessed June 3, 2025.

[13] Nakao Y.M., Gale C.P., Miyazaki K. (2023). Impact of a national screening programme on obesity and cardiovascular risk factors. Eur J Prev Cardiol.

[14] Ni Mhurchu C., Rodgers A., Pan W.H., Gu D.F., Woodward M., Asia Pacific Cohort Studies Collaboration (2004). Body mass index and cardiovascular disease in the Asia-Pacific Region: an overview of 33 cohorts involving 310 000 participants. Int J Epidemiol.

[15] Zheng W., McLerran D.F., Rolland B. (2011). Association between body-mass index and risk of death in more than 1 million Asians. N Engl J Med.

[16] Holmes M.V., Lange L.A., Palmer T. (2014). Causal effects of body mass index on cardiometabolic traits and events: a Mendelian randomization analysis. Am J Hum Genet.

[17] Hägg S., Fall T., Ploner A. (2015). Adiposity as a cause of cardiovascular disease: a Mendelian randomization study. Int J Epidemiol.

[18] Mongraw-Chaffin M.L., Peters S.A.E., Huxley R.R., Woodward M. (2015). The sex-specific association between BMI and coronary heart disease: a systematic review and meta-analysis of 95 cohorts with 1·2 million participants. Lancet Diabetes Endocrinol.

[19] Berrington de Gonzalez A., Hartge P., Cerhan J.R. (2010). Body-mass index and mortality among 1.46 million white adults. N Engl J Med.

[20] Hozawa A., Hirata T., Yatsuya H. (2019). Association between body mass index and all-cause death in Japanese population: pooled individual participant data analysis of 13 cohort studies. J Epidemiol.

[21] Marston N.A., Giugliano R.P., Im K. (2019). Association between triglyceride lowering and reduction of cardiovascular risk across multiple lipid-lowering therapeutic classes: a systematic review and meta-regression analysis of randomized controlled trials. Circulation.

[22] Castañer O., Pintó X., Subirana I. (2020). Remnant cholesterol, not LDL cholesterol, is associated with incident cardiovascular disease. J Am Coll Cardiol.

[23] Iso H., Imano H., Yamagishi K. (2014). Fasting and non-fasting triglycerides and risk of ischemic cardiovascular disease in Japanese men and women: the Circulatory Risk in Communities Study (CIRCS). Atherosclerosis.

[24] Wang A., Li Z., Zhou Y. (2014). Hypertriglyceridemic waist phenotype and risk of cardiovascular diseases in China: results from the Kailuan Study. Int J Cardiol.

[25] Toth P.P., Granowitz C., Hull M., Liassou D., Anderson A., Philip S. (2018). High triglycerides are associated with increased cardiovascular events, medical costs, and resource use: a real-world administrative claims analysis of statin-treated patients with high residual cardiovascular risk. J Am Heart Assoc.

[26] Cavero-Redondo I., Peleteiro B., Álvarez-Bueno C., Rodriguez-Artalejo F., Martínez-Vizcaíno V. (2017). Glycated haemoglobin A1c as a risk factor of cardiovascular outcomes and all-cause mortality in diabetic and non-diabetic populations: a systematic review and meta-analysis. BMJ Open.

[27] Schöttker B., Rathmann W., Herder C. (2016). HbA1c levels in non-diabetic older adults - No J-shaped associations with primary cardiovascular events, cardiovascular and all-cause mortality after adjustment for confounders in a meta-analysis of individual participant data from six cohort studies. BMC Med.

[28] Cooney M.T., Dudina A., De Bacquer D. (2009). HDL cholesterol protects against cardiovascular disease in both genders, at all ages and at all levels of risk. Atherosclerosis.

[29] Bowe B., Xie Y., Xian H., Balasubramanian S., Zayed M.A., Al-Aly Z. (2016). High density lipoprotein cholesterol and the risk of all-cause mortality among U.S. veterans. Clin J Am Soc Nephrol.

[30] Zhong G.C., Huang S.Q., Peng Y. (2020). HDL-C is associated with mortality from all causes, cardiovascular disease and cancer in a J-shaped dose-response fashion: a pooled analysis of 37 prospective cohort studies. Eur J Prev Cardiol.

