# Supplementary material for: Positive Impact of Health Check-Ups and Guidance in the General Population: A Database-Based Cohort Study in Japan
Source: AJPM Focus. 2025 Jun 17;4(4):100380. doi: 10.1016/j.focus.2025.100380 (PMC12275113; doi:10.1016/j.focus.2025.100380)
Supplement: Supplementary file 1 [file mmc1.pdf]

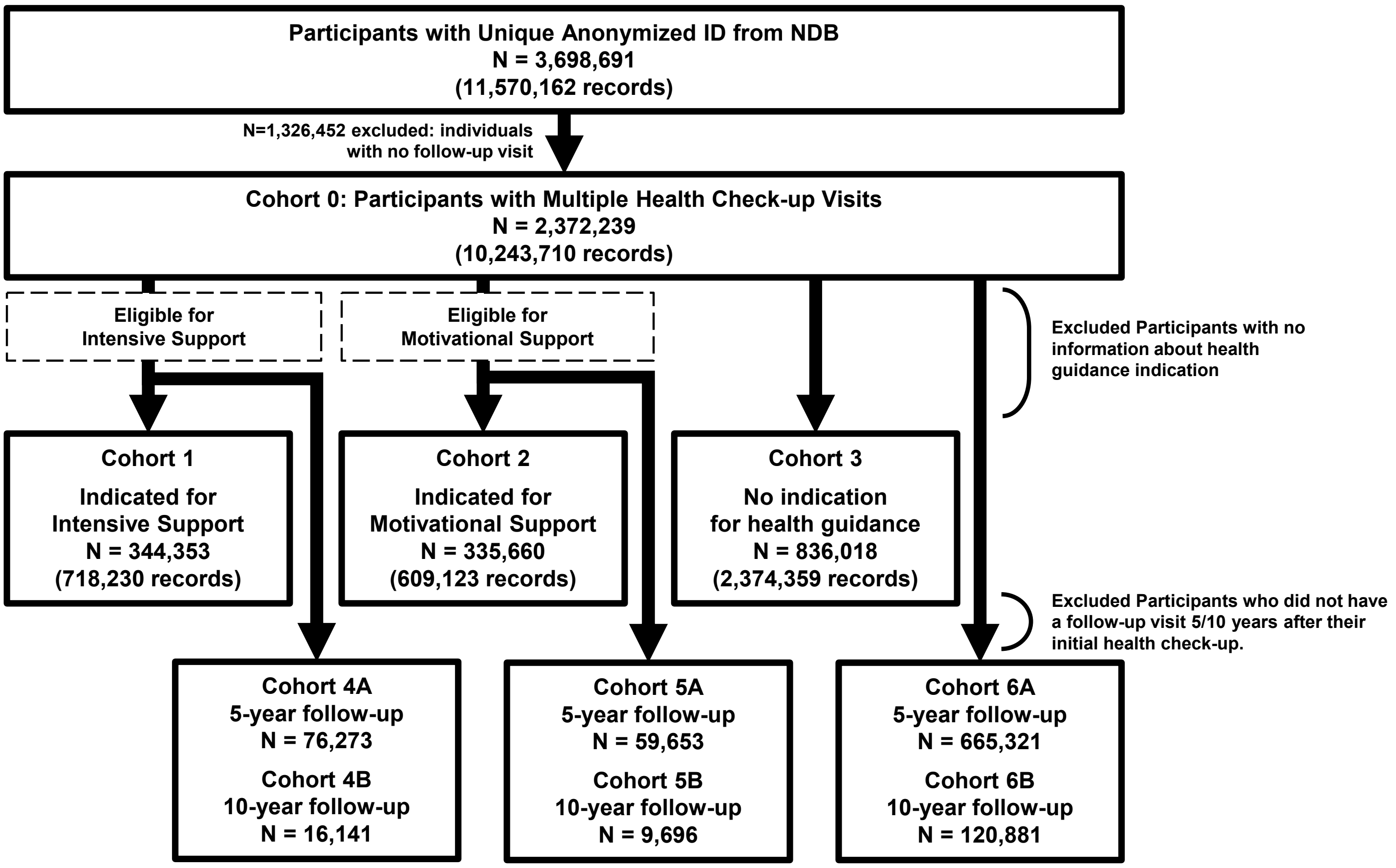

Supplementary Figure S1 (Appendix Figure 1)

A
Active Support: Males

B
Active Support: Females

C
Motivational Support: Males

D
Motivational Support: Females

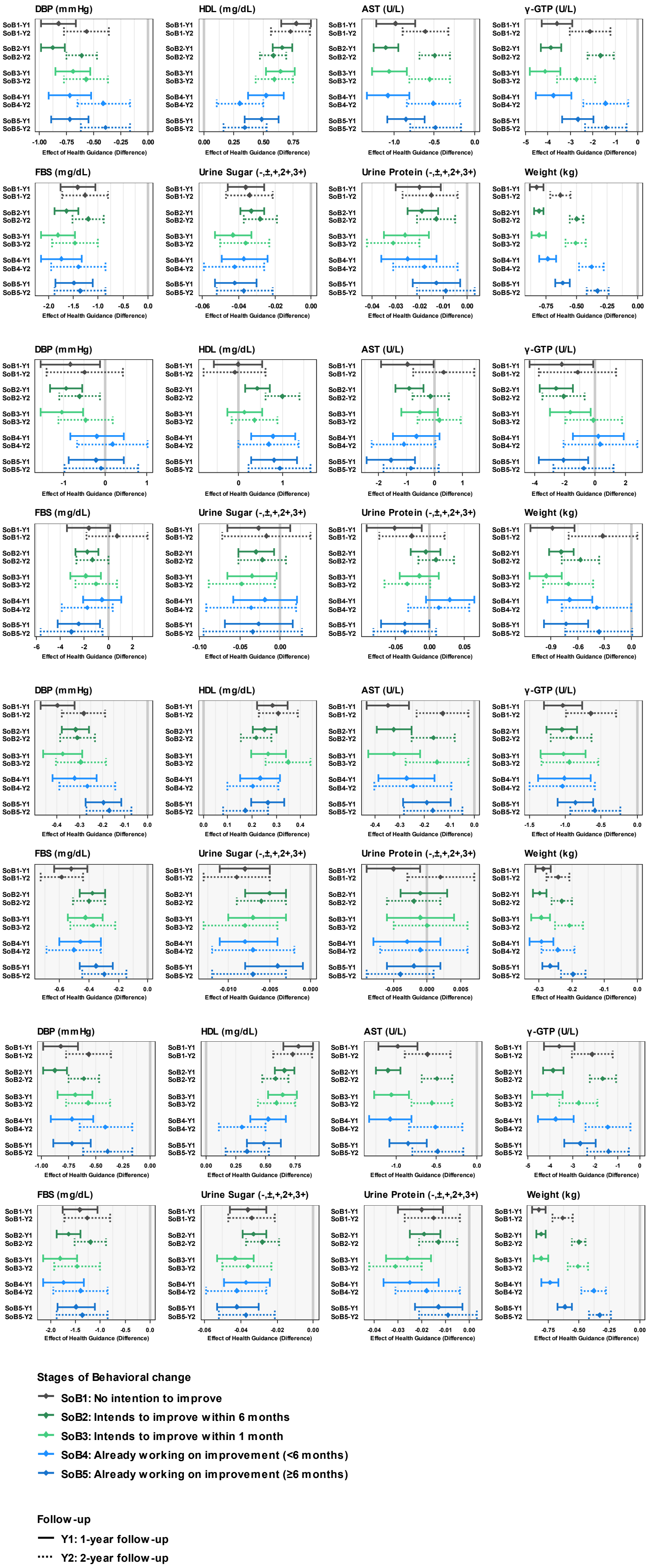

Supplementary Figure S2 (Appendix Figure 2)

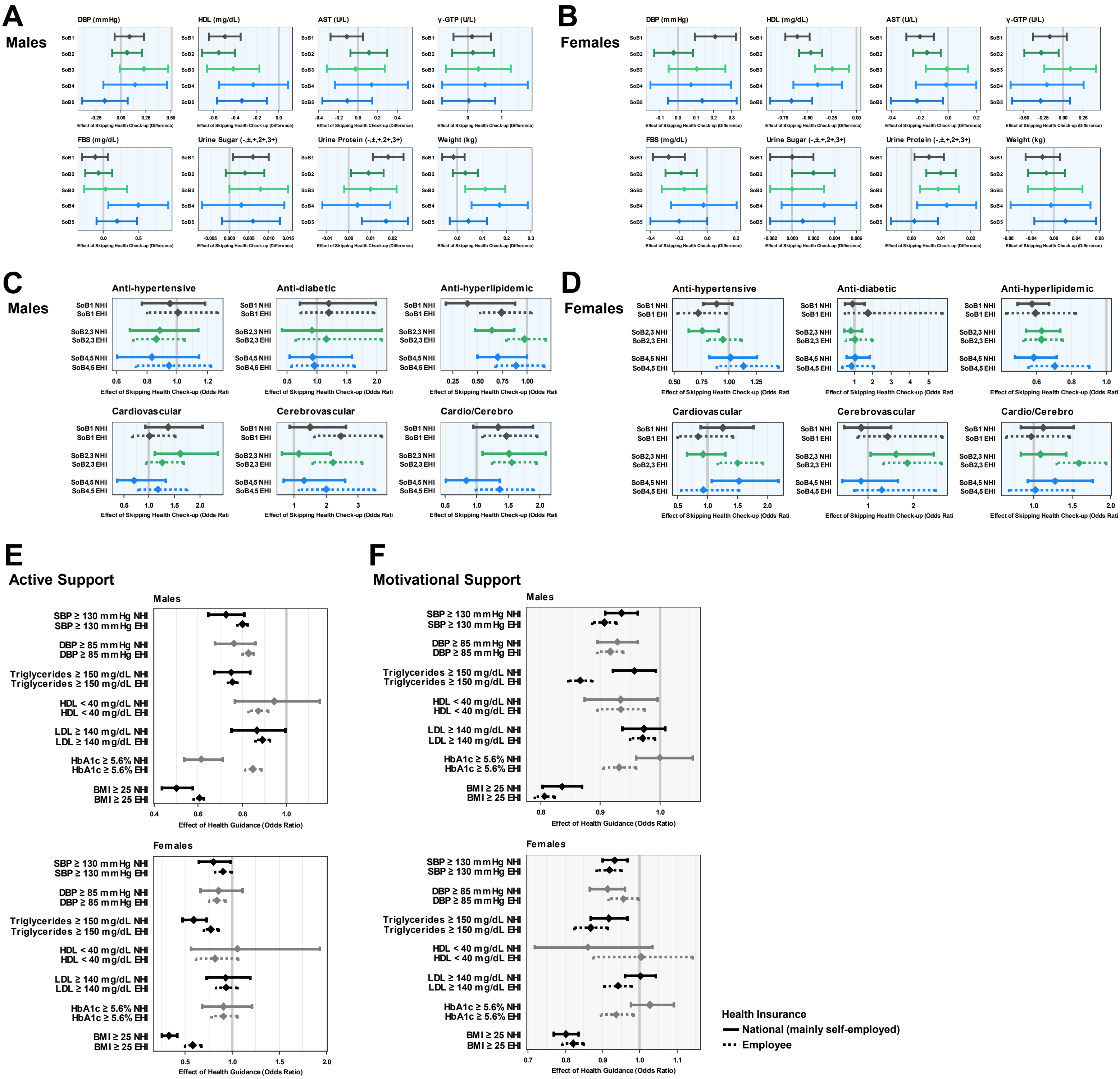

Supplementary Figure S3 (Appendix Figure 3)

A
Active Support: Males

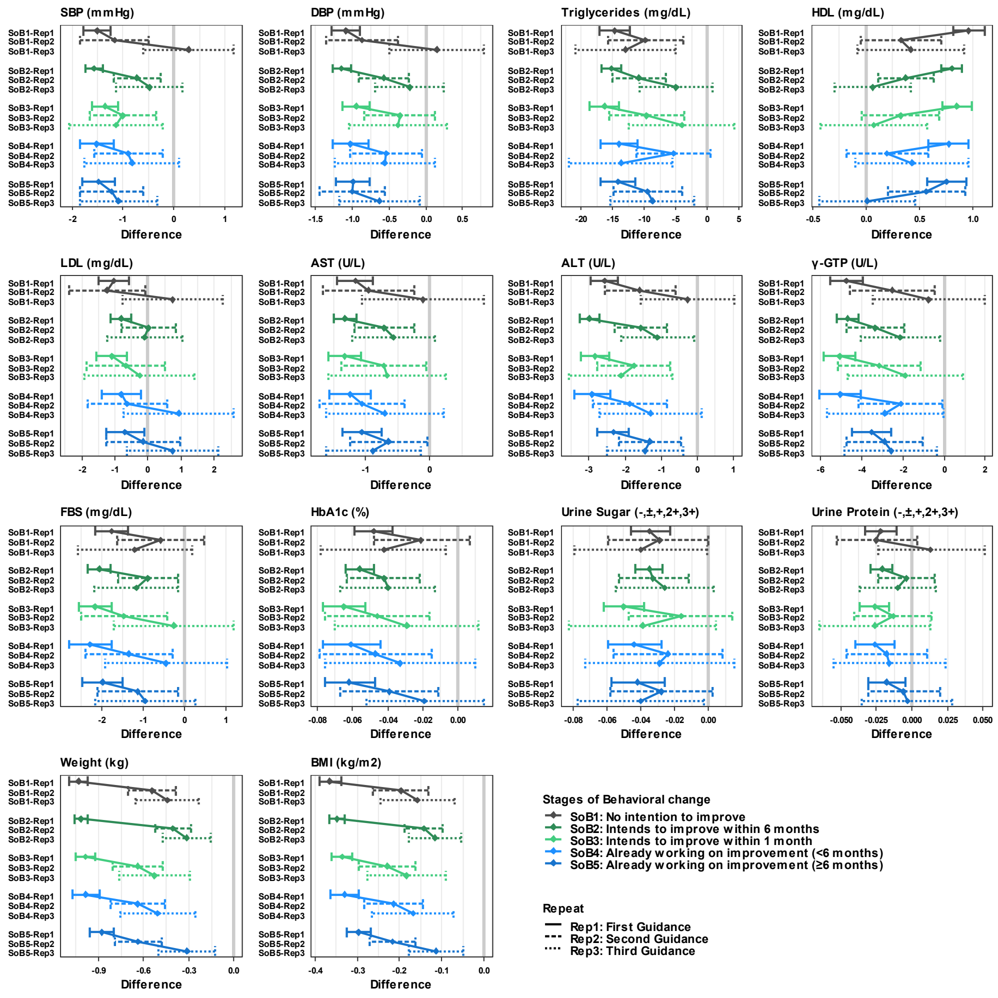

B
Active Support: Females

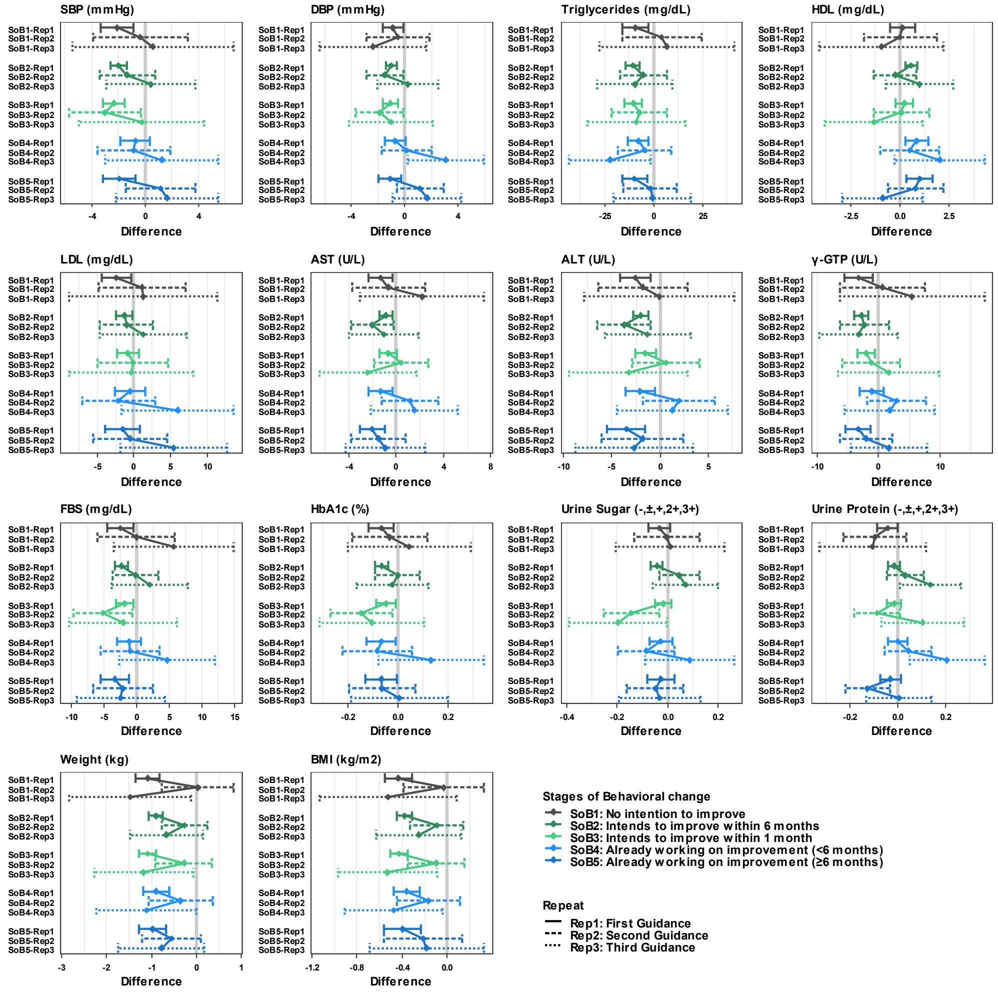

Supplementary Figure S4 (Appendix Figure 4)

A

Motivational Support: Males

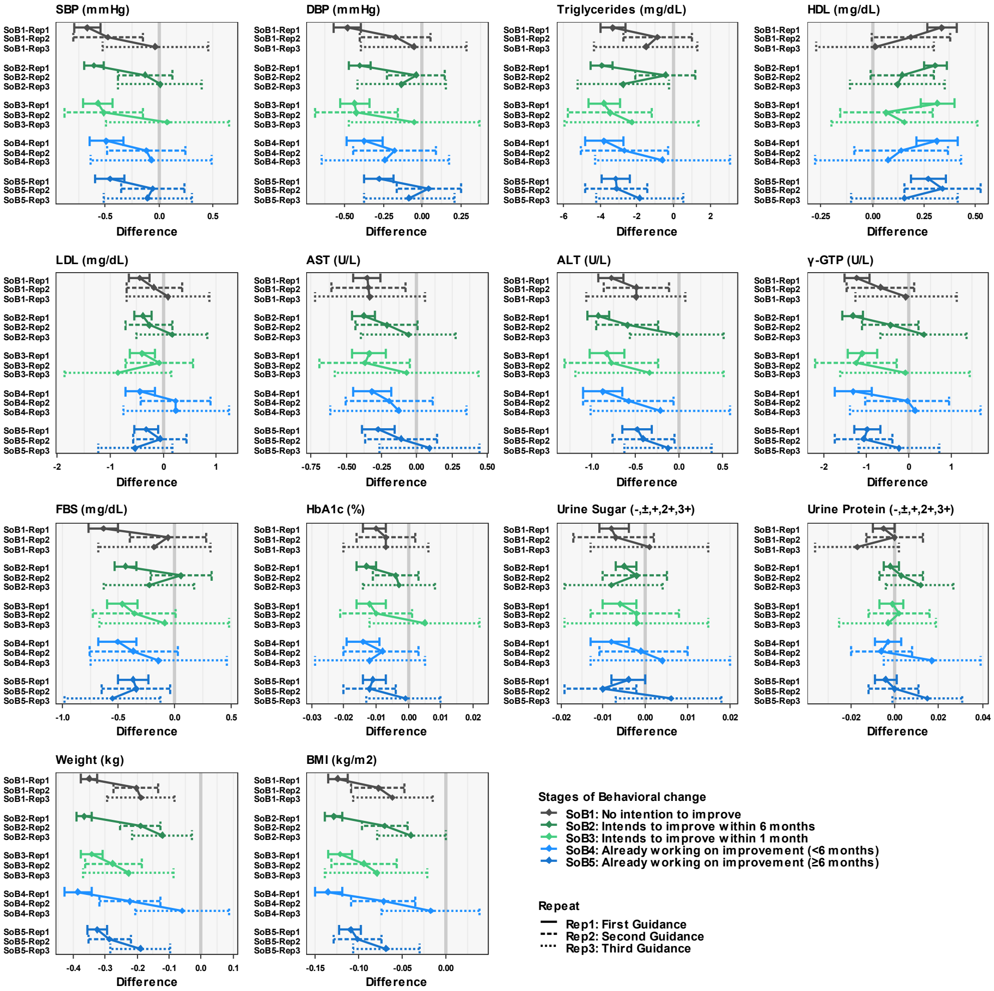

B

Motivational Support: Females

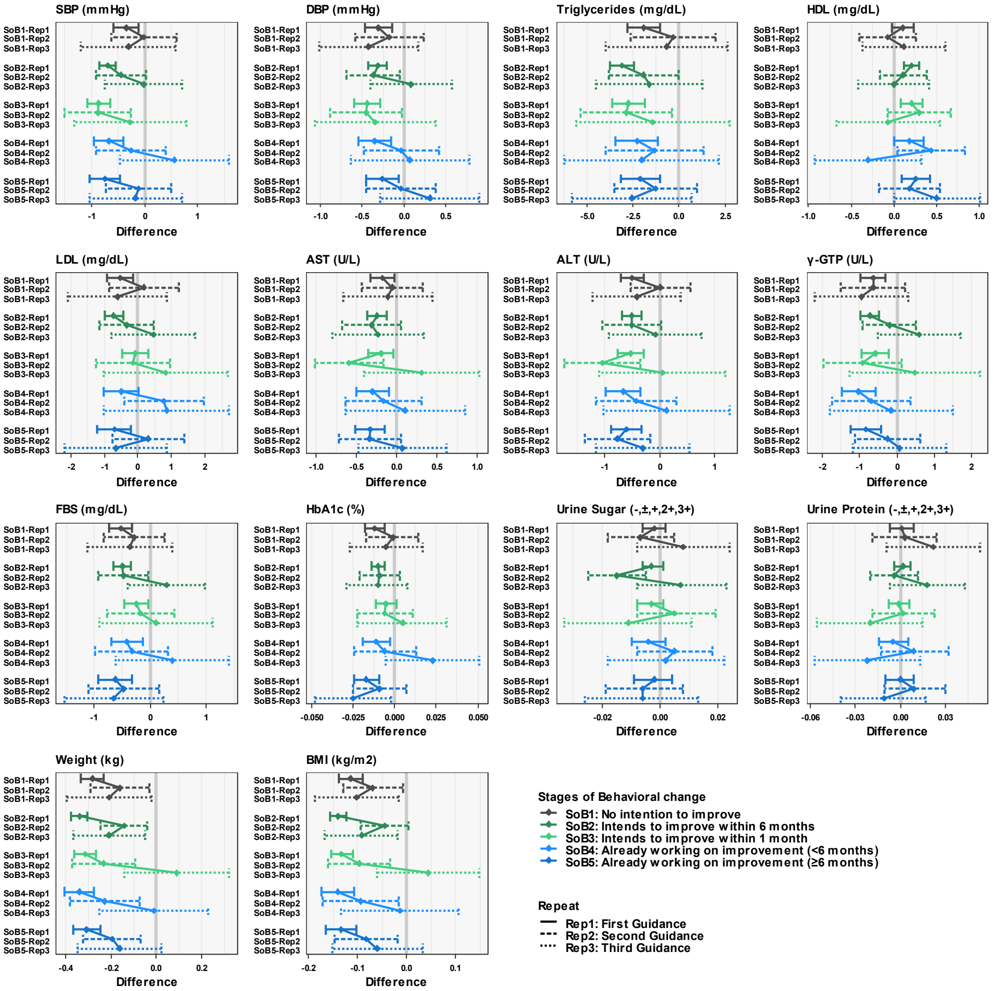

Supplementary Figure S5 (Appendix Figure 5)

A
Active Support: Males

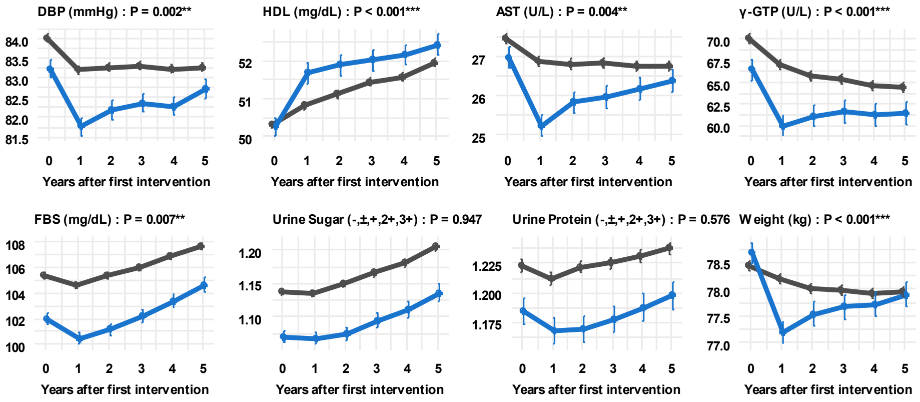

B
Active Support: Females

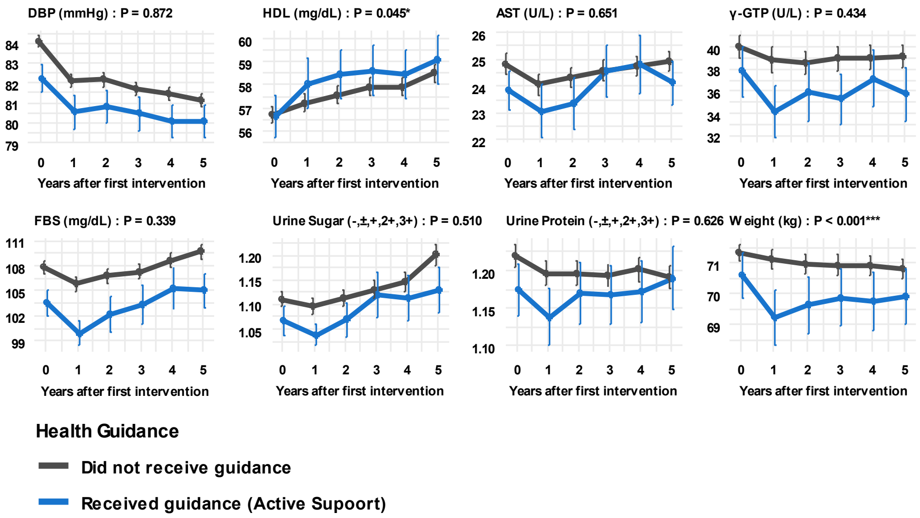

C
Motivational Support: Males

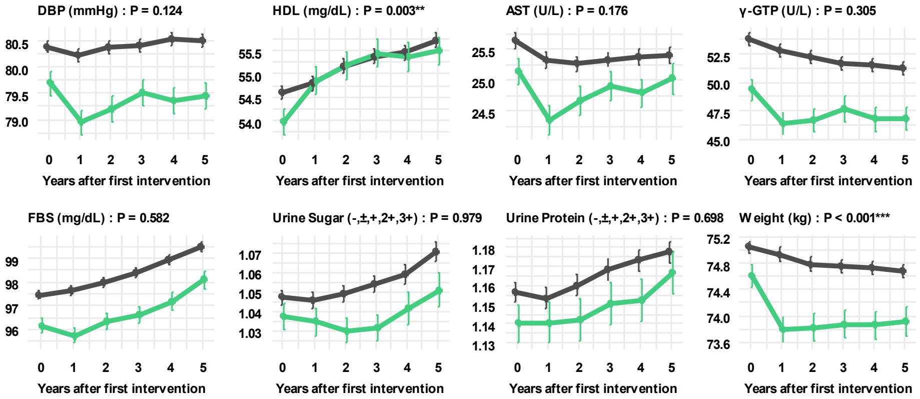

D
Motivational Support: Females

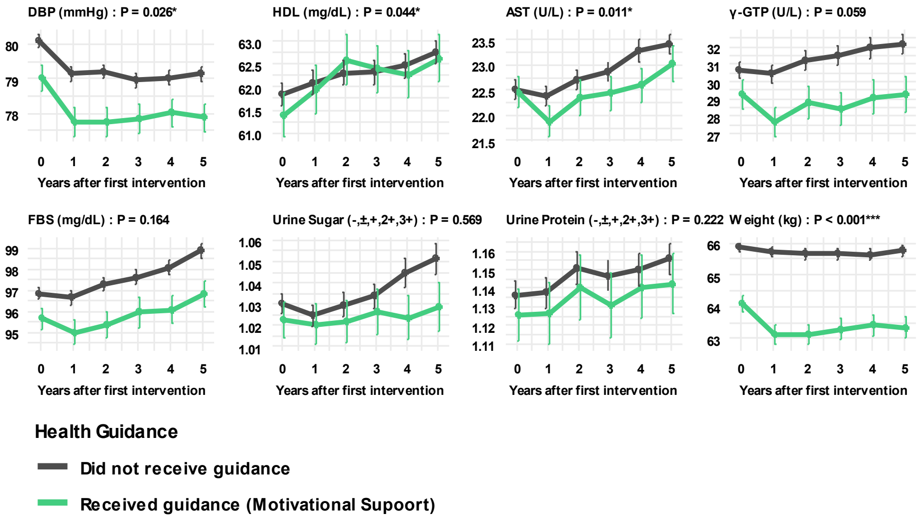

Supplementary Figure S6 (Appendix Figure 6)

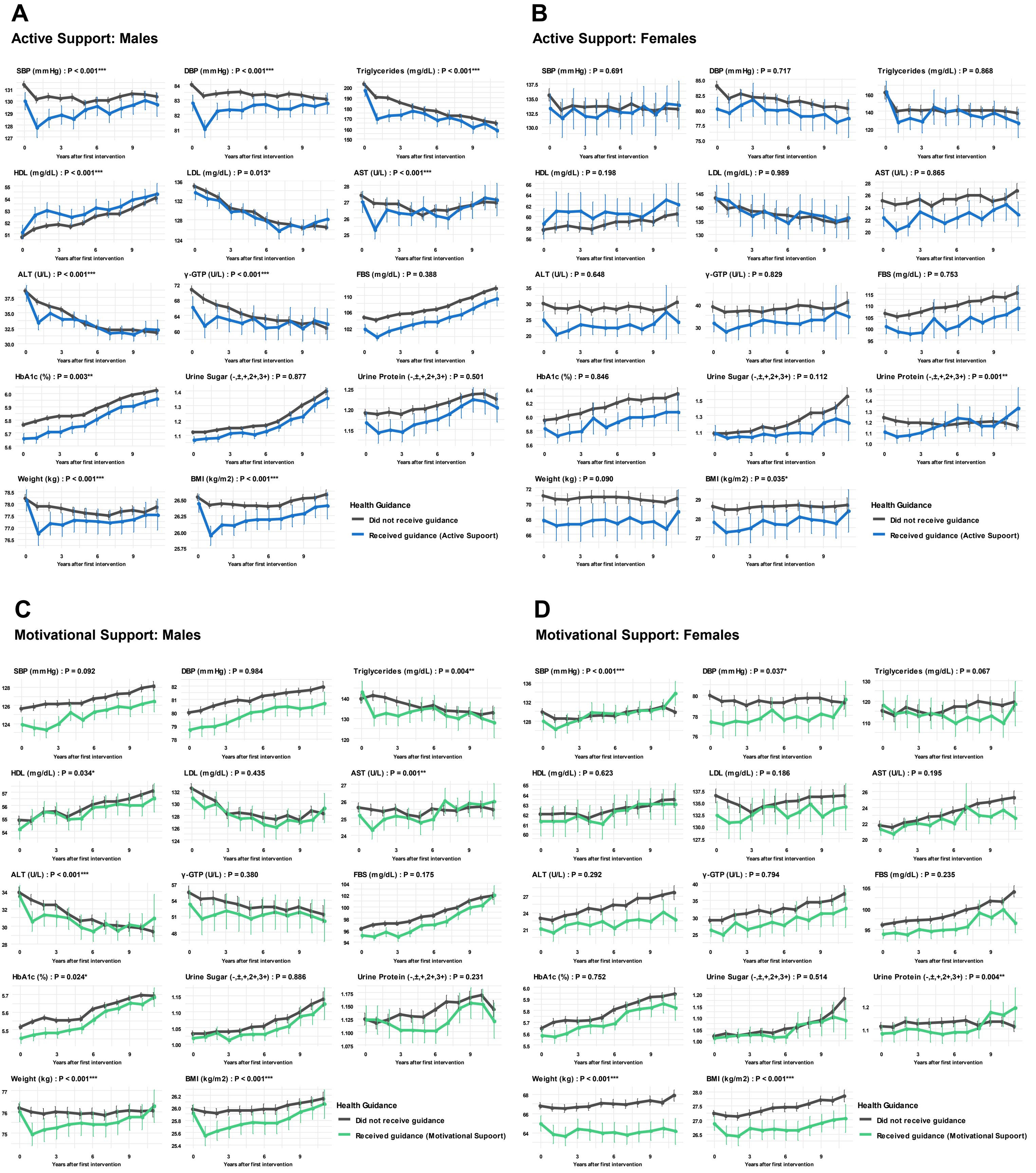

Supplementary Figure S7 (Appendix Figure 7)
