# Supplementary material for: Positive Impact of Health Check-Ups and Guidance in the General Population: A Database-Based Cohort Study in Japan
Source: AJPM Focus. 2025 Jun 17;4(4):100380. doi: 10.1016/j.focus.2025.100380 (PMC12275113; doi:10.1016/j.focus.2025.100380)
Supplement: Supplementary file 2 [file mmc2.docx]

| Appendix Table 1  Demographic characteristics of participants eligible for active support (Cohort 1) | | | | | | | | | | | | | |
| --- | --- | --- | --- | --- | --- | --- | --- | --- | --- | --- | --- | --- | --- |
| Sex | | | Males | | | | |  | Females | | | | |
| Received Health Guidance (active support) | | | No | |  | Yes | |  | No | |  | Yes | |
| Smoker | | | No | Yes |  | No | Yes |  | No | Yes |  | No | Yes |
| Health Guidance Receiving Rate (%) | | |  |  |  | 19.1% | |  |  |  |  | 14.9% | |
| Age (years) | | | 48.5 | 47.2 |  | 47.8 | 46.7 |  | 49.9 | 48.1 |  | 50.3 | 47.7 |
| N | | | 133935 | 152999 |  | 33062 | 34603 |  | 24662 | 10917 |  | 4548 | 1696 |
| Record Number | | | 237568 | 317262 |  | 44579 | 51060 |  | 38982 | 19320 |  | 5505 | 2166 |
| BMI (kg/m^2^) | | | 26.9 | 26.5 |  | 26.8 | 26.4 |  | 29.1 | 28.6 |  | 28.9 | 28.7 |
| Height (cm) | | | 171.4 | 171.7 |  | 171.7 | 172.0 |  | 157.6 | 158.2 |  | 157.6 | 158.5 |
| Weight (kg) | | | 79.1 | 78.0 |  | 79.0 | 78.2 |  | 72.3 | 71.7 |  | 71.9 | 72.1 |
| Abdominal Circumference (cm) | | | 93.0 | 92.3 |  | 92.7 | 91.9 |  | 96.6 | 95.5 |  | 96.6 | 95.8 |
| Systolic Blood Pressure (mmHg) | | | 135.7 | 129.2 |  | 133.8 | 127.6 |  | 138.8 | 130.8 |  | 136.8 | 129.4 |
| Diastolic Blood Pressure (mmHg) | | | 87.5 | 82.4 |  | 86.4 | 81.5 |  | 85.6 | 81.1 |  | 84.3 | 80.5 |
| Triglycerides (mg/dL) | | | 200.7 | 197.7 |  | 196.4 | 189.7 |  | 165.4 | 163.5 |  | 164.3 | 156.4 |
| HDL (mg/dL) | | | 51.7 | 49.6 |  | 51.7 | 49.9 |  | 57.6 | 56.2 |  | 57.2 | 56.5 |
| LDL (mg/dL) | | | 136.2 | 133.3 |  | 135.7 | 132.8 |  | 146.1 | 141.5 |  | 144.8 | 140.2 |
| Aspartate Aminotransferase (U/L) | | | 28.9 | 26.1 |  | 28.2 | 25.5 |  | 26.1 | 23.2 |  | 25.8 | 23.1 |
| Alanine Aminotransferase (U/L) | | | 39.0 | 34.7 |  | 38.2 | 33.9 |  | 31.0 | 26.6 |  | 30.7 | 26.4 |
| γ-Glutamyl Transpeptidase (U/L) | | | 71.8 | 66.4 |  | 68.2 | 63.3 |  | 41.8 | 40.8 |  | 41.3 | 38.9 |
| Fasting Blood Sugar (mg/dL) | | | 109.6 | 103.4 |  | 106.1 | 100.2 |  | 110.4 | 104.1 |  | 107.0 | 99.9 |
| Creatinine (mg/dL) | | | 0.88 | 0.84 |  | 0.88 | 0.85 |  | 0.63 | 0.63 |  | 0.65 | 0.64 |
| Hemoglobin A1c (%) | | | 5.86 | 5.76 |  | 5.74 | 5.66 |  | 6.03 | 5.85 |  | 5.92 | 5.75 |
| Smoker | Yes | | 53% | |  | 51% | |  | 31% | |  | 27% | |
| Exercise | > 30 min × two times per week | | 23% | 19% |  | 24% | 20% |  | 17% | 15% |  | 18% | 16% |
| Walking | > 1 hour per day | | 35% | 34% |  | 35% | 33% |  | 34% | 34% |  | 33% | 31% |
| Alcohol | Every day | | 37% | 40% |  | 34% | 37% |  | 12% | 22% |  | 12% | 22% |
|  | Sometimes | | 37% | 35% |  | 41% | 39% |  | 33% | 35% |  | 34% | 34% |
|  | Almost Never | | 26% | 26% |  | 25% | 24% |  | 54% | 43% |  | 54% | 44% |
| Stage of Behavioral Change | 1: No intention to improve | | 17% | 23% |  | 13% | 20% |  | 12% | 17% |  | 10% | 14% |
|  | 2: Intends to improve within 6 months | | 38% | 41% |  | 36% | 39% |  | 41% | 44% |  | 40% | 40% |
|  | 3: Intends to improve within 1 month | | 20% | 19% |  | 18% | 17% |  | 25% | 23% |  | 22% | 22% |
|  | 4: Already working on improvement (<6 months) | | 12% | 9% |  | 14% | 12% |  | 13% | 11% |  | 14% | 13% |
|  | 5: Already working on improvement (≥6 months) | | 13% | 8% |  | 19% | 13% |  | 9% | 6% |  | 14% | 12% |
| Health check-up outcomes in the year following being eligible for active support, with / without receiving support | | | | | | | | | | | | | |
| BMI (kg/m^2^) | | | 26.9 | 26.5 |  | 26.8 | 26.4 |  | 29.1 | 28.6 |  | 28.9 | 28.7 |
| Systolic Blood Pressure (mmHg) | | | 135.7 | 129.2 |  | 133.8 | 127.6 |  | 138.8 | 130.8 |  | 136.8 | 129.4 |
| Triglycerides (mg/dL) | | | 200.7 | 197.7 |  | 196.4 | 189.7 |  | 165.4 | 163.5 |  | 164.3 | 156.4 |
| LDL (mg/dL) | | | 136.2 | 133.3 |  | 135.7 | 132.8 |  | 146.1 | 141.5 |  | 144.8 | 140.2 |
| Alanine Aminotransferase (U/L) | | | 39.0 | 34.7 |  | 38.2 | 33.9 |  | 31.0 | 26.6 |  | 30.7 | 26.4 |
| Hemoglobin A1c (%) | | | 5.9 | 5.8 |  | 5.7 | 5.7 |  | 6.0 | 5.9 |  | 5.9 | 5.8 |
| BMI ≥ 25 kg/m^2^ | | | 70% | 66% |  | 64% | 62% |  | 87% | 85% |  | 81% | 81% |
| Systolic Blood Pressure ≥ 130 mmHg | | | 53% | 39% |  | 48% | 35% |  | 46% | 33% |  | 40% | 29% |
| Triglycerides ≥ 150 mg/dL | | | 52% | 52% |  | 46% | 47% |  | 39% | 41% |  | 35% | 34% |
| LDL ≥ 140 mg/dL | | | 43% | 40% |  | 41% | 39% |  | 54% | 49% |  | 52% | 47% |
| HbA1c ≥ 5.6% | | | 62% | 55% |  | 56% | 50% |  | 76% | 62% |  | 74% | 60% |
| New Medication | | Anti-hypertensive | 7.2% | 4.6% |  | 5.8% | 4.0% |  | 8.2% | 6.1% |  | 6.9% | 6.3% |
|  | | Anti-diabetic | 2.3% | 1.6% |  | 1.3% | 1.0% |  | 3.0% | 2.1% |  | 2.0% | 1.2% |
|  | | Anti-hyperlipidemic | 3.8% | 2.6% |  | 3.0% | 2.2% |  | 5.9% | 4.2% |  | 5.3% | 3.0% |
| Disease onset | | Cardiovascular | 0.5% | 0.5% |  | 0.4% | 0.4% |  | 0.5% | 0.4% |  | 0.4% | 0.1% |
|  | | Cerebrovascular | 1.2% | 1.1% |  | 1.0% | 1.0% |  | 0.8% | 0.8% |  | 0.8% | 0.5% |
|  | | Cadio or cerebrovascular | 1.6% | 1.5% |  | 1.4% | 1.3% |  | 1.3% | 1.1% |  | 1.1% | 0.6% |
| BMI, body mass index; HDL, high density lipoprotein; LDL, low density lipoprotein | | | | | | | | | | | | | |

| Appendix Table 2  Demographic characteristics of participants eligible for motivational support (Cohort 2) | | | | | | | | | | | | | | |
| --- | --- | --- | --- | --- | --- | --- | --- | --- | --- | --- | --- | --- | --- | --- |
| Sex | | | Males | | | | |  | | Females | | | | |
| Received Health Guidance (motivational support) | | | No | |  | Yes | | |  | No | |  | Yes | |
| Smoker | | | No | Yes |  | No | Yes | |  | No | Yes |  | No | Yes |
| Health Guidance Receiving Rate (%) | | |  |  |  | 24.4% | | |  |  |  |  | 22.2% | |
| Age (years) | | | 51.6 | 59.8 |  | 51.5 | 58.9 | |  | 53.5 | 52.7 |  | 56.1 | 53.9 |
| N | | | 185786 | 18496 |  | 60971 | 4909 | |  | 78590 | 5178 |  | 22843 | 1010 |
| Record Number | | | 316021 | 27145 |  | 86115 | 6173 | |  | 133342 | 7290 |  | 30737 | 1173 |
| BMI (kg/m2) | | | 25.8 | 25.1 |  | 25.7 | 25.1 | |  | 27.1 | 26.5 |  | 26.8 | 26.4 |
| Height (cm) | | | 170.4 | 167.1 |  | 170.5 | 167.1 | |  | 155.8 | 155.5 |  | 155.2 | 155.4 |
| Weight (kg) | | | 75.0 | 70.2 |  | 74.9 | 70.3 | |  | 65.9 | 64.1 |  | 64.6 | 63.6 |
| Abdominal Circumference (cm) | | | 90.4 | 88.1 |  | 90.1 | 87.6 | |  | 91.1 | 87.3 |  | 91.1 | 87.9 |
| Systolic Blood Pressure (mmHg) | | | 128.0 | 131.1 |  | 126.9 | 129.1 | |  | 131.4 | 127.4 |  | 130.4 | 126.4 |
| Diastolic Blood Pressure (mmHg) | | | 80.8 | 79.7 |  | 80.0 | 78.7 | |  | 80.0 | 77.6 |  | 78.8 | 76.6 |
| Triglycerides (mg/dL) | | | 136.1 | 158.4 |  | 135.7 | 154.7 | |  | 116.7 | 134.2 |  | 118.8 | 131.3 |
| HDL (mg/dL) | | | 55.3 | 52.0 |  | 55.1 | 51.8 | |  | 62.6 | 59.2 |  | 62.3 | 58.9 |
| LDL (mg/dL) | | | 132.1 | 129.4 |  | 131.7 | 129.0 | |  | 139.2 | 137.2 |  | 139.1 | 136.8 |
| Aspartate Aminotransferase (U/L) | | | 25.5 | 24.2 |  | 25.2 | 23.7 | |  | 22.8 | 21.2 |  | 22.7 | 20.8 |
| Alanine Aminotransferase (U/L) | | | 30.8 | 25.6 |  | 30.3 | 25.2 | |  | 23.3 | 21.3 |  | 22.6 | 20.5 |
| γ-Glutamyl Transpeptidase (U/L) | | | 51.9 | 53.4 |  | 49.8 | 49.4 | |  | 31.2 | 31.7 |  | 30.1 | 29.9 |
| Fasting Blood Sugar (mg/dL) | | | 98.0 | 101.6 |  | 96.8 | 98.8 | |  | 97.2 | 96.8 |  | 96.2 | 95.3 |
| Creatinine (mg/dL) | | | 0.9 | 0.9 |  | 0.9 | 0.8 | |  | 0.7 | 0.6 |  | 0.6 | 0.6 |
| Hemoglobin A1c (%) | | | 5.6 | 5.8 |  | 5.6 | 5.7 | |  | 5.7 | 5.7 |  | 5.7 | 5.7 |
| Smoker | Yes | | 9% | |  | 7% | | |  | 6% | |  | 4% | |
| Exercise | > 30 min × two times per week | | 30% | 34% |  | 32% | 37% | |  | 24% | 22% |  | 28% | 23% |
| Walking | > 1 hour per day | | 40% | 45% |  | 40% | 47% | |  | 40% | 41% |  | 40% | 41% |
| Alcohol | Every day | | 35% | 44% |  | 33% | 43% | |  | 11% | 20% |  | 10% | 18% |
|  | Sometimes | | 37% | 27% |  | 40% | 29% | |  | 32% | 33% |  | 31% | 31% |
|  | Almost Never | | 28% | 30% |  | 28% | 28% | |  | 57% | 48% |  | 59% | 50% |
| Stage of Behavioral Change | 1: No intention to improve | | 22% | 35% |  | 19% | 33% | |  | 17% | 22% |  | 19% | 21% |
|  | 2: Intends to improve within 6 months | | 34% | 31% |  | 32% | 31% | |  | 38% | 40% |  | 36% | 39% |
|  | 3: Intends to improve within 1 month | | 17% | 14% |  | 15% | 11% | |  | 21% | 20% |  | 18% | 17% |
|  | 4: Already working on improvement (<6 months) | | 11% | 8% |  | 13% | 9% | |  | 12% | 10% |  | 13% | 11% |
|  | 5: Already working on improvement (≥6 months) | | 16% | 12% |  | 20% | 17% | |  | 12% | 8% |  | 14% | 12% |
| Health check-up outcomes in the year following being eligible for motivational support, with / without receiving support | | | | | | | | | | | | | | |
| BMI (kg/m^2^) | | | 25.8 | 25.1 |  | 25.7 | 25.1 | |  | 27.1 | 26.5 |  | 26.8 | 26.4 |
| Systolic Blood Pressure (mmHg) | | | 128.0 | 131.1 |  | 126.9 | 129.1 | |  | 131.4 | 127.4 |  | 130.4 | 126.4 |
| Triglycerides (mg/dL) | | | 136.1 | 158.4 |  | 135.7 | 154.7 | |  | 116.7 | 134.2 |  | 118.8 | 131.3 |
| LDL (mg/dL) | | | 132.1 | 129.4 |  | 131.7 | 129.0 | |  | 139.2 | 137.2 |  | 139.1 | 136.8 |
| Alanine Aminotransferase (U/L) | | | 30.8 | 25.6 |  | 30.3 | 25.2 | |  | 23.3 | 21.3 |  | 22.6 | 20.5 |
| Hemoglobin A1c (%) | | | 5.6 | 5.8 |  | 5.6 | 5.7 | |  | 5.7 | 5.7 |  | 5.7 | 5.7 |
| BMI ≥ 25 kg/m^2^ | | | 60% | 54% |  | 55% | 51% | |  | 79% | 81% |  | 71% | 73% |
| Systolic Blood Pressure ≥ 130 mmHg | | | 34% | 28% |  | 30% | 24% | |  | 30% | 24% |  | 25% | 20% |
| Triglycerides ≥ 150 mg/dL | | | 30% | 39% |  | 27% | 37% | |  | 20% | 29% |  | 19% | 26% |
| LDL ≥ 140 mg/dL | | | 38% | 35% |  | 36% | 33% | |  | 46% | 44% |  | 45% | 42% |
| HbA1c ≥ 5.6% | | | 46% | 60% |  | 44% | 57% | |  | 60% | 55% |  | 61% | 57% |
| New Medication | | Anti-hypertensive | 4.8% | 7.3% |  | 3.7% | 6.1% | |  | 6.0% | 6.2% |  | 5.5% | 4.4% |
|  | | Anti-diabetic | 0.9% | 2.1% |  | 0.5% | 1.5% | |  | 1.0% | 1.2% |  | 0.7% | 0.4% |
|  | | Anti-hyperlipidemic | 2.8% | 4.2% |  | 2.2% | 3.5% | |  | 4.9% | 5.0% |  | 4.9% | 4.7% |
| Disease onset | | Cardiovascular | 0.5% | 1.0% |  | 0.5% | 0.8% | |  | 0.5% | 0.5% |  | 0.5% | 0.3% |
|  | | Cerebrovascular | 1.4% | 1.9% |  | 1.1% | 1.6% | |  | 1.1% | 1.0% |  | 1.0% | 0.8% |
|  | | Cadio or cerebrovascular | 1.8% | 2.7% |  | 1.5% | 2.2% | |  | 1.5% | 1.5% |  | 1.5% | 1.0% |
| BMI, body mass index; HDL, high density lipoprotein; LDL, low density lipoprotein | | | | | | | | | | | | | | |

| Appendix Table 3  Demographic characteristics of participants not eligible for health guidance (Cohort 3) | | | | | | | | | | | | |
| --- | --- | --- | --- | --- | --- | --- | --- | --- | --- | --- | --- | --- |
| Sex | | Males | | | | |  | Females | | | | |
| Stage of Behavioral Change (1 to 5) | | 1 | 2 | 3 | 4 | 5 |  | 1 | 2 | 3 | 4 | 5 |
| Age (years) | | 53.3 | 50.2 | 50.2 | 50.7 | 53.8 |  | 56.2 | 51.6 | 51.7 | 53.1 | 57.1 |
| N | | 511187 | 652204 | 412432 | 297900 | 368630 |  | 396493 | 538814 | 347891 | 233171 | 260546 |
| Record Number | | 1292930 | 1493855 | 676577 | 431469 | 795868 |  | 993562 | 1204466 | 572338 | 329520 | 512665 |
| BMI (kg/m2) | | 23.0 | 24.3 | 24.7 | 24.8 | 23.8 |  | 21.3 | 22.3 | 22.7 | 22.8 | 21.9 |
| Height (cm) | | 169.2 | 170.1 | 170.2 | 170.3 | 169.6 |  | 155.5 | 156.8 | 156.9 | 156.7 | 155.8 |
| Weight (kg) | | 65.9 | 70.5 | 71.6 | 72.0 | 68.7 |  | 51.5 | 54.8 | 55.8 | 56.0 | 53.1 |
| Abdominal Circumference (cm) | | 82.7 | 86.2 | 87.0 | 87.0 | 84.3 |  | 77.3 | 79.6 | 80.5 | 80.9 | 79.0 |
| Systolic Blood Pressure (mmHg) | | 124.9 | 125.2 | 125.6 | 125.3 | 125.3 |  | 120.7 | 118.9 | 119.3 | 120.0 | 121.4 |
| Diastolic Blood Pressure (mmHg) | | 77.6 | 78.9 | 79.5 | 79.0 | 78.1 |  | 72.4 | 72.4 | 73.0 | 73.0 | 73.0 |
| Triglycerides (mg/dL) | | 122.5 | 141.9 | 143.9 | 136.9 | 120.9 |  | 87.4 | 90.9 | 92.2 | 93.2 | 90.2 |
| HDL (mg/dL) | | 60.3 | 56.9 | 56.2 | 56.2 | 60.0 |  | 72.1 | 70.5 | 69.6 | 69.0 | 71.7 |
| LDL (mg/dL) | | 120.6 | 125.7 | 126.4 | 125.6 | 122.1 |  | 121.5 | 123.7 | 125.1 | 125.9 | 125.0 |
| Aspartate Aminotransferase (U/L) | | 24.1 | 25.3 | 25.7 | 25.2 | 24.2 |  | 21.8 | 21.4 | 21.6 | 21.8 | 22.3 |
| Alanine Aminotransferase (U/L) | | 24.3 | 29.3 | 30.1 | 28.9 | 25.0 |  | 17.6 | 18.6 | 19.1 | 19.3 | 18.8 |
| γ-Glutamyl Transpeptidase (U/L) | | 48.3 | 55.5 | 56.4 | 51.8 | 45.6 |  | 24.8 | 26.0 | 26.3 | 26.0 | 24.9 |
| Fasting Blood Sugar (mg/dL) | | 98.0 | 100.3 | 101.9 | 101.6 | 102.0 |  | 91.6 | 92.4 | 93.2 | 93.5 | 93.8 |
| Creatinine (mg/dL) | | 0.9 | 0.9 | 0.9 | 0.9 | 0.9 |  | 0.6 | 0.6 | 0.6 | 0.7 | 0.7 |
| Hemoglobin A1c (%) | | 5.6 | 5.7 | 5.7 | 5.7 | 5.7 |  | 5.6 | 5.6 | 5.6 | 5.6 | 5.7 |
| Smoker | Yes | 37% | 37% | 34% | 27% | 21% |  | 11% | 12% | 11% | 9% | 6% |
| Exercise | > 30 min × two times per week | 29% | 16% | 21% | 32% | 61% |  | 32% | 15% | 19% | 31% | 63% |
| Walking | > 1 hour per day | 42% | 32% | 36% | 47% | 61% |  | 49% | 36% | 40% | 51% | 67% |
| Alcohol | Every day | 43% | 39% | 37% | 34% | 36% |  | 16% | 15% | 14% | 13% | 12% |
|  | Sometimes | 29% | 35% | 37% | 39% | 37% |  | 27% | 33% | 33% | 34% | 30% |
|  | Almost Never | 28% | 26% | 26% | 26% | 27% |  | 57% | 52% | 53% | 53% | 58% |
| BMI, body mass index; HDL, high density lipoprotein; LDL, low density lipoprotein | | | | | | | | | | | | |

| Appendix Table 4 | | | | | | | | | | | | | | | | | | | | | | | | | | | | | | |
| --- | --- | --- | --- | --- | --- | --- | --- | --- | --- | --- | --- | --- | --- | --- | --- | --- | --- | --- | --- | --- | --- | --- | --- | --- | --- | --- | --- | --- | --- | --- |
| Demographic characteristics of participants not eligible for health guidance, by next year’s check-up attendance | | | | | | | | | | | | | | | | | | | | | | | | | | | | | | |
| Sex | | Male | | | | | | | | | | | | | |  | Female | | | | | | | | | | | | | |
| Stages of Behavioral Change (1 to 5) | | 1 | | | 2 | | | 3 | | | 4 | | | 5 | |  | 1 | | | 2 | | | 3 | | | 4 | | | 5 | |
| Skipping Next Year's Check-up | | No | Yes |  | No | Yes |  | No | Yes |  | No | Yes |  | No | Yes |  | No | Yes |  | No | Yes |  | No | Yes |  | No | Yes |  | No | Yes |
| Age (year) | | 49.5 | 51.1 |  | 47.0 | 47.6 |  | 47.1 | 47.7 |  | 47.4 | 48.6 |  | 50.2 | 51.1 |  | 52.4 | 53.2 |  | 48.5 | 48.3 |  | 48.6 | 48.1 |  | 49.8 | 49.5 |  | 53.6 | 53.1 |
| N | | 169793 | 12058 |  | 182224 | 11344 |  | 98198 | 4609 |  | 67359 | 2677 |  | 96908 | 4998 |  | 151334 | 18932 |  | 198176 | 21459 |  | 115704 | 10327 |  | 71679 | 5272 |  | 80461 | 6884 |
| Record Number | | 357735 | 12842 |  | 335875 | 11825 |  | 138222 | 4699 |  | 84792 | 2725 |  | 170896 | 5180 |  | 314236 | 20932 |  | 381414 | 22962 |  | 171725 | 10773 |  | 92583 | 5387 |  | 135498 | 7227 |
| BMI (kg/m2) | | 21.6 | 21.7 |  | 22.3 | 22.3 |  | 22.6 | 22.6 |  | 22.8 | 22.7 |  | 22.3 | 22.3 |  | 20.4 | 20.5 |  | 21.1 | 21.1 |  | 21.4 | 21.4 |  | 21.5 | 21.5 |  | 20.8 | 20.9 |
| Height (cm) | | 169.8 | 169.3 |  | 170.4 | 170.0 |  | 170.4 | 170.2 |  | 170.6 | 170.2 |  | 170.1 | 169.7 |  | 156.6 | 156.3 |  | 157.6 | 157.5 |  | 157.7 | 157.6 |  | 157.6 | 157.5 |  | 156.8 | 156.8 |
| Weight (kg) | | 62.4 | 62.1 |  | 64.8 | 64.5 |  | 65.6 | 65.4 |  | 66.4 | 66.0 |  | 64.6 | 64.3 |  | 50.0 | 50.2 |  | 52.4 | 52.4 |  | 53.2 | 53.2 |  | 53.3 | 53.4 |  | 51.1 | 51.4 |
| Abdominal Circumference (cm) | | 78.3 | 78.7 |  | 80.2 | 80.5 |  | 80.8 | 80.9 |  | 81.2 | 81.3 |  | 79.6 | 79.7 |  | 74.6 | 74.9 |  | 76.2 | 76.4 |  | 77.0 | 77.1 |  | 77.3 | 77.5 |  | 75.8 | 76.0 |
| Systolic Blood Pressure (mmHg) | | 119.5 | 120.9 |  | 118.7 | 119.5 |  | 118.8 | 119.4 |  | 118.7 | 119.4 |  | 119.8 | 120.3 |  | 115.7 | 117.4 |  | 113.9 | 114.1 |  | 114.0 | 114.0 |  | 114.3 | 114.9 |  | 116.1 | 116.6 |
| Diastolic Blood Pressure (mmHg) | | 74.9 | 75.4 |  | 74.8 | 75.1 |  | 75.2 | 75.6 |  | 74.9 | 75.0 |  | 75.2 | 75.1 |  | 70.4 | 71.0 |  | 70.0 | 69.9 |  | 70.3 | 70.1 |  | 70.2 | 70.4 |  | 70.7 | 70.9 |
| Triglyceride (mg/dL) | | 101.9 | 106.0 |  | 110.5 | 114.0 |  | 111.2 | 113.5 |  | 106.7 | 109.9 |  | 98.6 | 101.3 |  | 77.4 | 80.1 |  | 78.3 | 78.9 |  | 79.0 | 79.3 |  | 79.6 | 80.7 |  | 78.7 | 80.5 |
| HDL (mg/dL) | | 63.5 | 62.6 |  | 60.8 | 60.5 |  | 60.2 | 60.6 |  | 60.1 | 59.8 |  | 63.9 | 63.3 |  | 74.3 | 73.4 |  | 72.8 | 72.2 |  | 72.0 | 71.4 |  | 71.8 | 71.2 |  | 74.5 | 74.3 |
| LDL (mg/dL) | | 118.7 | 119.8 |  | 123.4 | 124.7 |  | 124.5 | 124.5 |  | 124.8 | 126.4 |  | 122.5 | 123.6 |  | 119.8 | 122.7 |  | 120.3 | 120.7 |  | 121.8 | 122.1 |  | 123.4 | 123.9 |  | 124.8 | 125.0 |
| Aspartate Aminotransferase (U/L) | | 22.6 | 22.9 |  | 22.8 | 23.2 |  | 23.0 | 23.5 |  | 22.8 | 23.1 |  | 22.7 | 22.9 |  | 20.8 | 21.0 |  | 20.1 | 20.2 |  | 20.2 | 20.2 |  | 20.5 | 20.6 |  | 21.4 | 21.4 |
| Alanine Aminotransferase (U/L) | | 21.4 | 21.5 |  | 23.7 | 24.1 |  | 24.2 | 24.4 |  | 23.6 | 23.4 |  | 21.7 | 22.0 |  | 16.3 | 16.6 |  | 16.3 | 16.6 |  | 16.5 | 16.7 |  | 16.8 | 17.0 |  | 17.1 | 17.2 |
| γ-Glutamyl Transpeptidase (U/L) | | 40.7 | 42.6 |  | 44.5 | 47.1 |  | 45.3 | 46.7 |  | 42.0 | 43.5 |  | 38.8 | 39.6 |  | 22.6 | 22.9 |  | 22.6 | 22.5 |  | 22.7 | 22.6 |  | 22.6 | 23.0 |  | 22.4 | 22.7 |
| Fasting Blood Sugar (mg/dL) | | 93.3 | 93.7 |  | 93.6 | 93.8 |  | 94.1 | 94.2 |  | 93.8 | 94.1 |  | 94.5 | 94.8 |  | 89.1 | 89.0 |  | 89.4 | 89.1 |  | 89.7 | 89.5 |  | 89.8 | 89.7 |  | 89.9 | 89.7 |
| Creatinine (mg/dL) | | 0.9 | 0.8 |  | 0.9 | 0.9 |  | 0.9 | 0.9 |  | 0.9 | 0.6 |  | 0.9 | 0.9 |  | 0.6 | 0.6 |  | 0.6 | 0.6 |  | 0.6 | 0.6 |  | 0.6 | 0.6 |  | 0.7 | 0.7 |
| Hemoglobin A1c (%) | | 5.4 | 5.5 |  | 5.4 | 5.5 |  | 5.4 | 5.4 |  | 5.4 | 5.5 |  | 5.5 | 5.5 |  | 5.5 | 5.5 |  | 5.4 | 5.4 |  | 5.4 | 5.4 |  | 5.5 | 5.5 |  | 5.5 | 5.5 |
| Smoker | Yes | 39% | 41% |  | 39% | 43% |  | 35% | 38% |  | 28% | 29% |  | 21% | 23% |  | 12% | 10% |  | 12% | 11% |  | 11% | 10% |  | 9% | 8% |  | 7% | 7% |
| Exercise | > 30 min x 2-time per week | 29% | 29% |  | 15% | 14% |  | 21% | 20% |  | 30% | 32% |  | 62% | 63% |  | 29% | 30% |  | 13% | 13% |  | 17% | 17% |  | 27% | 27% |  | 61% | 60% |
| Walking | > 1 hour per day | 42% | 43% |  | 32% | 33% |  | 37% | 36% |  | 46% | 48% |  | 60% | 61% |  | 47% | 49% |  | 34% | 36% |  | 39% | 40% |  | 49% | 51% |  | 65% | 67% |
| Alcohol | Everyday | 41% | 44% |  | 38% | 41% |  | 37% | 39% |  | 34% | 38% |  | 36% | 37% |  | 18% | 17% |  | 16% | 16% |  | 15% | 14% |  | 14% | 14% |  | 14% | 14% |
|  | Sometimes | 31% | 28% |  | 36% | 34% |  | 38% | 35% |  | 41% | 38% |  | 40% | 37% |  | 30% | 28% |  | 35% | 34% |  | 36% | 35% |  | 37% | 37% |  | 34% | 34% |
|  | Almost Never | 28% | 28% |  | 26% | 25% |  | 26% | 26% |  | 25% | 25% |  | 25% | 27% |  | 52% | 55% |  | 48% | 50% |  | 49% | 51% |  | 48% | 49% |  | 53% | 52% |
| Health check-up outcomes after two years, with / without skipping next year’s check-ups | | | | | | | | | | | | | | | | | | | | | | | | | | | | | | |
| BMI (kg/m2) | | 21.6 | 21.7 |  | 22.3 | 22.3 |  | 22.6 | 22.6 |  | 22.8 | 22.7 |  | 22.3 | 22.3 |  | 20.4 | 20.5 |  | 21.1 | 21.1 |  | 21.4 | 21.4 |  | 21.5 | 21.5 |  | 20.8 | 20.9 |
| Systolic Blood Pressure (mmHg) | | 119.5 | 120.9 |  | 118.7 | 119.5 |  | 118.8 | 119.4 |  | 118.7 | 119.4 |  | 119.8 | 120.3 |  | 115.7 | 117.4 |  | 113.9 | 114.1 |  | 114.0 | 114.0 |  | 114.3 | 114.9 |  | 116.1 | 116.6 |
| Triglycerides (mg/dL) | | 101.9 | 106.0 |  | 110.5 | 114.0 |  | 111.2 | 113.5 |  | 106.7 | 109.9 |  | 98.6 | 101.3 |  | 77.4 | 80.1 |  | 78.3 | 78.9 |  | 79.0 | 79.3 |  | 79.6 | 80.7 |  | 78.7 | 80.5 |
| LDL (mg/dL) | | 118.7 | 119.8 |  | 123.4 | 124.7 |  | 124.5 | 124.5 |  | 124.8 | 126.4 |  | 122.5 | 123.6 |  | 119.8 | 122.7 |  | 120.3 | 120.7 |  | 121.8 | 122.1 |  | 123.4 | 123.9 |  | 124.8 | 125.0 |
| Alanine Aminotransferase (U/L) | | 21.4 | 21.5 |  | 23.7 | 24.1 |  | 24.2 | 24.4 |  | 23.6 | 23.4 |  | 21.7 | 22.0 |  | 16.3 | 16.6 |  | 16.3 | 16.6 |  | 16.5 | 16.7 |  | 16.8 | 17.0 |  | 17.1 | 17.2 |
| Hemoglobin A1c (%) | | 5.4 | 5.5 |  | 5.4 | 5.5 |  | 5.4 | 5.4 |  | 5.4 | 5.5 |  | 5.5 | 5.5 |  | 5.5 | 5.5 |  | 5.4 | 5.4 |  | 5.4 | 5.4 |  | 5.5 | 5.5 |  | 5.5 | 5.5 |
| BMI ≥ 25 kg/m2 | | 7% | 8% |  | 13% | 14% |  | 15% | 15% |  | 18% | 18% |  | 12% | 12% |  | 4% | 4% |  | 9% | 9% |  | 10% | 11% |  | 11% | 11% |  | 6% | 7% |
| Systolic Blood Pressure ≥ 130 mmHg | | 26% | 30% |  | 23% | 25% |  | 24% | 26% |  | 24% | 26% |  | 26% | 28% |  | 21% | 25% |  | 17% | 18% |  | 17% | 17% |  | 18% | 20% |  | 22% | 23% |
| Triglycerides ≥ 150 mg/dL | | 15% | 17% |  | 19% | 21% |  | 20% | 21% |  | 20% | 22% |  | 15% | 16% |  | 6% | 7% |  | 6% | 7% |  | 6% | 7% |  | 7% | 8% |  | 6% | 7% |
| LDL ≥ 140 mg/dL | | 25% | 26% |  | 30% | 30% |  | 31% | 30% |  | 32% | 33% |  | 30% | 31% |  | 26% | 29% |  | 28% | 28% |  | 29% | 30% |  | 31% | 32% |  | 32% | 33% |
| HbA1c ≥ 5.6% | | 33% | 38% |  | 32% | 34% |  | 32% | 33% |  | 34% | 36% |  | 36% | 39% |  | 41% | 46% |  | 36% | 37% |  | 37% | 36% |  | 39% | 39% |  | 43% | 44% |
| New Medication | Anti-hypertensive | 2.7% | 3.4% |  | 2.6% | 2.9% |  | 2.7% | 2.5% |  | 2.8% | 2.9% |  | 3.0% | 3.1% |  | 2.8% | 2.9% |  | 2.1% | 2.0% |  | 2.2% | 1.8% |  | 2.4% | 2.3% |  | 2.6% | 2.9% |
|  | Anti-diabetic | 0.4% | 0.6% |  | 0.5% | 0.7% |  | 0.5% | 0.6% |  | 0.7% | 0.7% |  | 0.9% | 1.0% |  | 0.2% | 0.3% |  | 0.2% | 0.2% |  | 0.2% | 0.2% |  | 0.3% | 0.2% |  | 0.4% | 0.5% |
|  | Anti-hyperlipidemic | 1.6% | 1.6% |  | 1.9% | 2.0% |  | 2.1% | 1.8% |  | 2.4% | 2.5% |  | 2.5% | 2.0% |  | 3.2% | 2.7% |  | 2.7% | 2.2% |  | 2.9% | 2.0% |  | 3.6% | 2.8% |  | 4.3% | 3.2% |
| Disease onset | Cardiovascular | 0.7% | 0.8% |  | 0.7% | 1.0% |  | 0.7% | 0.9% |  | 0.8% | 1.5% |  | 0.9% | 0.7% |  | 0.5% | 0.6% |  | 0.5% | 0.6% |  | 0.5% | 0.5% |  | 0.6% | 0.6% |  | 0.7% | 0.7% |
|  | Cerebrovascular | 0.3% | 0.5% |  | 0.3% | 0.5% |  | 0.3% | 0.4% |  | 0.3% | 0.5% |  | 0.3% | 0.5% |  | 0.2% | 0.2% |  | 0.2% | 0.2% |  | 0.2% | 0.4% |  | 0.3% | 0.5% |  | 0.3% | 0.2% |
|  | Cadio or cerebrovascular | 1.0% | 1.3% |  | 1.0% | 1.4% |  | 1.0% | 1.4% |  | 1.0% | 1.9% |  | 1.2% | 1.2% |  | 0.7% | 0.8% |  | 0.7% | 0.8% |  | 0.7% | 0.9% |  | 0.8% | 1.1% |  | 0.9% | 0.9% |
