# Supplementary material for: Positive Impact of Health Check-Ups and Guidance in the General Population: A Database-Based Cohort Study in Japan
Source: AJPM Focus. 2025 Jun 17;4(4):100380. doi: 10.1016/j.focus.2025.100380 (PMC12275113; doi:10.1016/j.focus.2025.100380)
Supplement: Supplementary file 3 [file mmc3.docx]

**Appendix Methods**

**Additional information for the study population**

The aim was to acquire data for about 1 million people that might offer sufficient statistical power for various subgroup analyses and research hypothesis testing, and which was convenient for data handling. This figure is based on an estimated health examination participation rate of about 30%, aiming for a total population of approximately 4 million people. Cities and wards in the Kanto region with geographical representation were selected while considering geographical balance. Preference was given to selecting mainly a single city for efficiency in data extraction and merging processes. Outside the metropolitan areas, large cities at a certain distance from the city center were chosen, while for metropolitan areas, wards located relatively centrally and with a larger population size were selected. The cities and wards are as follows: Saitama City (Saitama prefecture), Chiba City (Chiba prefecture), Hachioji City, Hino City, Tachikawa City (Tama area in Tokyo prefecture), Koto Ward, Shinagawa Ward, and Shinjuku Ward (Central area in Tokyo prefecture). Due to the nature of the database, reasons for loss of follow-up cannot be obtained. Possible reasons may include scheduling conflicts, relocation, hospitalization, death, or non-attendance without specific reasons (such as underestimating the necessity or lacking awareness of health improvement). All participants included in the study underwent at least two annual health check-ups. The receipt of Specific Health Checkups and Health Guidance is confirmed by the existence of records, each created for a separate visit and marked with a unique visit ID in addition to the personal ID. Eligibility for Specific Health Guidance is indicated within these records by a specific field.

**Additional information on definitions of the cohorts**

All cohorts are included in Cohort 0, from which they are filtered according to various criteria. Cohort 1 consisted of participants eligible for active support, Cohort 2 consisted of those eligible for motivational support, and Cohort 3 included participants not eligible for either. The outcomes for Cohorts 1 to 3 included physical measurements, laboratory data, new prescriptions, and new disease onset one or two years after becoming eligible for specific health guidance. Cohort 3 excluded any participants who (1) had records indicating the use of antihypertensive, antidiabetic, or antihyperlipidemic medications, or (2) had a past history of cerebrovascular disease, cardiovascular disease, or renal failure. Cohorts 4 and 5 consisted of participants who were eligible for health guidance at their first health check-up (Active: Cohort 4, Motivational: Cohort 5) and were subsequently followed up for 5 (Cohort 4A/5A) and 10 (Cohort 4B/5B) years. The outcomes for Cohorts 4 and 5 were trends in physical measurements and laboratory data over 5- and 10-year follow-up periods after the first health check-up. Cohort 6 included all participants, irrespective of risk level, who completed follow-up periods of 5 (6A) and 10 (6B) years. The outcomes for Cohort 6 were physical measurements and laboratory data at specific health check-up visits 5- and 10-years after the first visit. Participants in Cohorts 4 to 6 had to have health check-up visits exactly 5 and 10 years after their initial visit to be included.

**Appendix Figure Legends**

**Appendix Figure 1. Cohorts’ Composition**

Overview of study participants by cohort assignment indicating numbers and records for each group. ID, identification; NDB, National Database of Health Insurance Claims and Specific Health Checkups of Japan

**Appendix Figure 2. Effectiveness of Health Guidance on Changes in Other Metabolic Syndrome Indicators**

Model-based effectiveness of receiving health guidance among eligible participants stratified by stage of behavioral change. Differences in continuous outcomes (excluding those assessed in Figure 1) between those who received support with those who did not after a 1-year and 2-year follow-up are shown by solid and dotted lines, respectively.

A and B: Changes in metabolic syndrome indicators for males (A) and females (B) who received active support. C and D: The outcomes for males (C) and females (D) who received motivational support. AST, aspartate aminotransferase; DBP, diastolic blood pressure; FBS, fasting blood sugar; GTP, glutamyl transpeptidase; HDL, high density lipoprotein; SoB, Stage of behavioral change

**Appendix Figure 3. Effect of Skipping Health Check-ups on Changes in Metabolic Syndrome Indicators**

Model-based effectiveness of skipping health check-ups among healthy participants ineligible for health guidance and stratified by stage of behavioral change. Differences in continuous outcomes (A and B) and odds ratios for binary outcomes at a health check-up two years later (C to F). Analyses in C to F were stratified by the type of health insurance. The comparison was between participants who skipped their health check-up in the intervening year and those who did not. A and B: Regarding weight and BMI (in Figure 3A/3B), some correlations were observed in men, with the group in stages 3 to 4 of behavioral change showing 0.1–0.2 kg greater weight and 0.03–0.06 higher BMI. In stage 1 of behavioral change, a minimal effect was observed for weight and BMI. This may be related to the fact that individuals in stage 1 tend not to be overweight and therefore have less potential for weight reduction compared to those in stages 2 to 4. C to F: Effect on the risk of new medications and new onset of cardi-cerebrovascular diseases, as well as exceeding certain cut-offs for metabolic indicators stratified by the type of health insurance. AST, aspartate aminotransferase; BMI, body mass index; DBP, diastolic blood pressure; EHI, employee health insurance; FBS, fasting blood sugar; GTP, glutamyl transpeptidase; HbA1c, hemoglobin A1c; HDL, higher density lipoprotein; LDL, low density lipoprotein; NHI, national health insurance; SBP, systolic blood pressure; SoB, Stage of behavioral change

**Appendix Figure 4. Effect of Previous Health Guidance History on Current Effectiveness (Active Support)**

Model-based estimates of the effectiveness of repeatedly receiving health guidance among participants eligible for health guidance and stratified by stage of behavioral change. Differences in continuous outcomes between those who received support and those who did not receive support for the first, second, and third time are shown by solid, dashed, and dotted lines, respectively. ALT, alanine aminotransferase; AST, aspartate aminotransferase; BMI, body mass index; DBP, diastolic blood pressure; FBS, fasting blood sugar; GTP, glutamyl transpeptidase; HbA1c, hemoglobin A1c; HDL, higher density lipoprotein; LDL, low density lipoprotein; SBP, systolic blood pressure; SoB, Stage of behavioral change

A and B: Changes in metabolic syndrome indicators for males (A) and females (B)

**Appendix Figure 5. Effect of Previous Health Guidance History on Current Effectiveness (Motivational Support)**

Model-based estimates of the effectiveness of receiving health guidance repeatedly among participants eligible for health guidance stratified by the stage of behavioral change. Differences in continuous outcomes between those who received support and those who did not receive support for the first, second, and third time are shown by solid, dashed, and dotted lines, respectively. ALT, alanine aminotransferase; AST, aspartate aminotransferase; BMI, body mass index; DBP, diastolic blood pressure; FBS, fasting blood sugar; GTP, glutamyl transpeptidase; HbA1c, hemoglobin A1c; HDL, higher density lipoprotein; LDL, low density lipoprotein; SBP, systolic blood pressure; SoB, Stage of behavioral change

A and B: Changes in metabolic syndrome indicators for males (A) and females (B)

**Appendix 6. Effect of Health Guidance on Other Metabolic Syndrome Indicators Over Time**

Longitudinal tracking of health outcomes comparing participants who received health guidance and those who did not.

A and B: Changes in metabolic syndrome indicators (excluding those assessed in Figure 4) over five years for males (A) and females (B) who received active support. C and D: Outcomes for males (C) and females (D) who received motivational support. AST, aspartate aminotransferase; DBP, diastolic blood pressure; FBS, fasting blood sugar; GTP, glutamyl transpeptidase; HDL, higher density lipoprotein

**Appendix 7. Effect of Health Guidance on Metabolic Syndrome Indicators Over 10 Years**

Longitudinal tracking of health outcomes comparing participants who received health guidance and those who did not.

A and B: Changes in metabolic syndrome indicators over ten years for males (A) and females (B) who received active support. C and D: Outcomes for males (C) and females (D) who received motivational support. ALT, alanine aminotransferase; AST, aspartate aminotransferase; BMI, body mass index; DBP, diastolic blood pressure; FBS, fasting blood sugar; GTP, glutamyl transpeptidase; HbA1c, hemoglobin A1c; HDL, higher density lipoprotein; LDL, low density lipoprotein; SBP, systolic blood pressure
